# Effects of forest structure on the interaction between avian hosts, dipteran vectors and haemosporidian parasites

**DOI:** 10.1186/s12898-020-00315-5

**Published:** 2020-08-19

**Authors:** Willem van Hoesel, Diego Santiago-Alarcon, Alfonso Marzal, Swen C. Renner

**Affiliations:** 1 Institute of Zoology, University of Natural Resources and Life Sciences, Gregor-Mendel Straße 33, 1180 Vienna, Austria; 2 Instituto de Ecología, Red de Biología Y Conservación de Vertebrados, Coatepec 351 El Haya, 91070 Xalapa, Veracruz Mexico; 3 Department of Zoology, University of Extremadura, Avenida de Elvas s/n, 06006 Badajoz, Spain; 4 Ornithology, Natural History Museum Vienna, Burggasse 7, 1010 Vienna, Austria

**Keywords:** Avian malaria, Ecosystem parasitology, Haemosporida, Land use, Landscape epizootiology, Ceratopogonidae, Culicidae, Simuliidae

## Abstract

**Background:**

Forest habitats are important biodiversity refuges for a wide variety of bird species. Parasitism may modulate host species presence and abundance, and parasite effects can change according to forest management practices. Such processes are not well studied in vector-borne avian haemosporidians. We analyzed the effects of forest management on bird-dipteran-haemosporidian interactions, using seven common bird species in managed and unmanaged beech forest habitats in northeastern Germany. We assumed that forest structural heterogeneity affects parasite population parameters in avian hosts (i.e., prevalence and parasitemia), through its effect on the condition of the avian host but also through varying vector abundances.

**Results:**

Parasite prevalence was high (about 80%) and homogeneous across different beech forest categories (i.e., young, old, unmanaged) and for all bird species, except *Erithacus rubecula* (35%). Parasitemia varied across bird species but not across forest categories within each avian species (lowest parasitemia were found in *E*. *rubecula*, *Turdus merula*, and *Turdus philomelos*). In our study system, we found that vector abundance was not the main driver of parasite dynamics. We found that forest structure affects parasite infection probability directly and potentially host condition via available resources that have to be used either to combat infections (i.e., high parasitemia) or to maintain a good body condition.

**Conclusions:**

The effects of each of the predictors were bird species-specific, and we found that Diptera vectors were not the foremost influence in our host-vector-parasite system. Effects of forest habitat variables indicated that for most bird species in this study, habitat regulation of infection probability was more likely (i.e., *E. rubecula*, *Fringilla coelebs*, *Sylvia atricapilla*), whereas for *Parus major* habitat characteristics impacted first individuals' body condition and subsequently the probability of infection. Our findings emphasize the need of species-specific analyses and to use continuous forest structural parameters (e.g., the proportion of gap, south facing aspect) to better understand habitat and land use effects on host-vector-parasite dynamics.

## Background

Parasites are an essential component of natural systems and ecosystem processes through their effects on host individuals and thus populations [1]. Parasites typically have negative effects on hosts and may also affect hosts' inter- and intraspecific interactions [2]. Host physiological changes due to an infection can potentially change host behavior [3], fitness, reproductive success, or survival [4]. Chronic infections produce recurring disease symptoms that may adversely affect hosts throughout their life [5], regulating host populations and modifying interactions within communities [6]. Bottom-up and top-down processes are likely in host-parasite systems, specifically when the environment either influences first the parasites (top-down regulation) or the host physical condition (bottom-up regulation) [7]. So far, bottom-up and top-down processes have been suggested [2] but not demonstrated for host-vector-parasite interactions in a varying environment. Therefore, our understanding of the functioning of natural communities will increase by studying the relative importance of both regulatory forces within the same community.

Three-quarters of all bird species use forest habitats [8]. Increasingly, habitats are subjected to human alteration, resulting in habitat degradation, fragmentation and diversity loss through land use intensification [9]. Forest modifications are particularly driven by forestry and management regimes [10], where local species richness is affected through forest structural heterogeneity and/or forest type [11]. Forest habitats provide resources for avian hosts, such as nesting sites, shelter, foraging opportunities and food [12]. However, not all forest types are optimal for all bird species, and there are species-specific preferences of habitat resources [13].

Habitat conditions and resource availability may have direct effects on host fitness [14]. The effects on fitness are reflected in body growth and condition [15], and also in the birds' ability to invest in immune responses to fight-off pathogens (e.g. [16]). Outcomes of this trade-off can be observed in bilateral asymmetries, such as that of tarsus growth, which reflect early developmental challenges that depend on available resources of the place the young birds inhabit [17]. The life history and habitat preferences of the avian hosts play an essential role in determining the likelihood of an infection [6, 18]. Hence, habitat change alters ecosystem processes and species interactions, leading for example to higher infection rates in more intensively used habitats [19, 20].

Haemosporidian parasites are intracellular parasites that affect a wide range of vertebrate species. These parasites are transmitted by dipterans of different families in which the sexual reproduction takes place. Vertebrate hosts are then required for the asexual phase of the parasite's reproduction [21]. The effects of an infection vary greatly among host species and can lead to chronic lifelong infections (e.g. [22]). Many effects on individuals are non-lethal, such as weight loss and anemia [21], decreased reproduction [23], changes in body asymmetries [24], or inhibited feather growth [17]. In addition to the fitness effects, parasite infections acting in synergy with anthropogenic modifications of the environment can cause increased infection risk and changes in local abundance of hosts and vectors (e.g. [25, 26]).

Studies incorporating all three components in the interaction between blood parasites, avian hosts and their insect vectors are scarce (e.g. [27]). Similarly to the avian hosts, vectors may be affected by structural differences in forest habitats or forest management [20]. This ultimately affects vector distribution and abundance, subsequently influencing exposure to parasites and transmission risk [28, 29], changing the spatial variation of prevalence in hosts [30]. Forest habitat structure and management may therefore affect host-vector-parasite interactions in a predictable way, as a function of both avian and insect abundances. In a previous study, the forest habitat was included explicitly as forest categories (i.e., forest stands dominated by one tree species, such as beech or spruce) and forest structure (i.e., continuous variables assessing the interior structural heterogeneity, such as amount of gaps, canopy heterogeneity and canopy height), but the vectors have not been explicitly included in the models so far [19].

The original hypotheses (c.f. [19]) can be interpreted as a 'top-down'-hypothesis and a 'bottom-up'-hypothesis. In the top-down-hypothesis, forest structure (as a proxy for management) a priori determines bird infection risk due to the habitat suitability for vectors, in turn triggering an immune response in birds, which then consumes resources fighting-off the infection that would otherwise be available for body growth (measured by asymmetries in tarsus and feather growth). We regard this hypothesis as top-down regulation from the perspective of the avian host, since the parasite effects determine the bird's condition. In the bottom-up-hypothesis, development of the avian host is primarily affected by the forest structure through the availability of resources in the given habitat [31]. Suboptimal habitats provide fewer resources to invest in immune response and defending against parasite infections; the host needs to invest more energy in foraging to compensate for the detrimental effects of habitat quality. Here, there is bottom-up regulation from the perspective of the avian hosts, since the hosts' body condition determines infection risk. In this study, we extend the original hypotheses in two ways:

First, we extend the number of avian host species from two to seven species, which will improve our understanding of the relationship between host traits on parasitism (e.g., the prevalence of avian haemosporidians has been shown to be positively related to the abundance of hosts species; [32]).

Second, because vectors are known to be affected by forest habitat structure [20], we explicitly add dipteran vectors as an important mechanistic driver for the variation and spread of avian haemosporidians [28, 33]. Based on the importance of vector abundance for the transmission of avian haemosporidians [27], we finally hypothesize that the amended models would better explain the host-vector-parasite interactions, compared to the previous study in which the models only included bird hosts and forest structure [19].

We approach the analysis of our data using two different statistical methods: through model selection and Structural Equation Models (SEMs). The usage of SEMs is novel approach in the field of host-vector-parasite interaction [19, 34], and allows the analysis of multiple pathways and associations among variables, thus offering a way to improve our understanding of host-vector-parasite interactions. We explain our statistical approach in more detail in “Methods” section.

## Results

We captured 498 individuals of seven avian host species over two years. Parasite status was analyzed for 441 individuals using microscopy, and 403 individuals through Polymerase Chain Reaction (PCR). Either method resulted in a parasite prevalence of 65% (respectively 288 out of 441 and 260 out of 403 individuals). For 398 individuals we could determine the status through both methods, resulting in 80% infection prevalence over the seven avian host species (Table 1). In 71% of all infected individuals both methods yielded a positive result for the same individual, indicating 29% discrepancy between microscopy and PCR methods. The total prevalence over all avian host species is higher using a combination of both microscopy and the PCR methods (80%).

**Table 1:** Overview of parasite prevalence and parasitemia per avian host species within each forest category

Species	Forest category	Parasite prevalence (mean)	CI (lower)	CI (upper)	*n*	Parasite intensity (median)	CI (lower)	CI (upper)	*n*
*Cyanistes caeruleus*	Unmanaged	0.88	0.25	0.13	8	1.16	0.38	0.64	8
	Age class—old	0.82	0.18	0.18	17	0.93	0.63	1.25	16
	Age class—young	0.78	0.33	0.22	9	0.85	0.85	1.34	9
*Erithacus rubecula*	Unmanaged	0.47	0.21	0.21	19	na	na	0.30	19
	Age class—old	0.35	0.15	0.19	26	na	na	0.30	25
	Age class—young	0.28	0.17	0.17	29	na	na	na	28
*Fringilla coelebs*	Unmanaged	0.92	0.12	0.08	25	1.32	0.42	0.29	25
	Age class—old	0.91	0.18	0.09	11	0.95	0.48	0.87	11
	Age class—young	0.75	0.38	0.25	8	0.60	0.60	1.09	6
*Parus major*	Unmanaged	0.83	0.25	0.17	12	1.04	0.74	0.68	11
	Age class—old	0.82	0.11	0.11	44	0.74	0.44	0.44	44
	Age class—young	0.90	0.13	0.10	30	1.31	0.50	0.10	30
*Sylvia atricapilla*	Unmanaged	0.80	0.40	0.20	5	0.60	0.60	0.96	5
	Age class—old	0.97	0.07	0.03	29	0.90	0.13	0.28	29
	Age class—young	0.93	0.11	0.07	28	1.10	0.27	0.25	28
*Turdus merula*	Unmanaged	1.00	0.12	na	18	0.54	0.24	0.48	18
	Age class—old	1.00	0.12	na	16	0.60	0.30	0.24	16
	Age class—young	0.96	0.08	0.04	25	0.70	0.40	0.30	25
*Turdus philomelos*	Unmanaged	1.00	na	na	8	0.30	na	0.48	8
	Age class—old	0.88	na	na	16	0.30	na	na	16
	Age class—young	0.87	na	na	15	0.30	na	0.30	15

Parasite prevalence (mean) and parasitemia (median, of log_10_ x + 1 transformed data) per avian host species within each forest category. Confidence intervals (CI) are given with level of 95% after 5000 bootstrap replications, "na" indicates that the CI is not available (i.e., was not calculated)

**Fig. 1:**
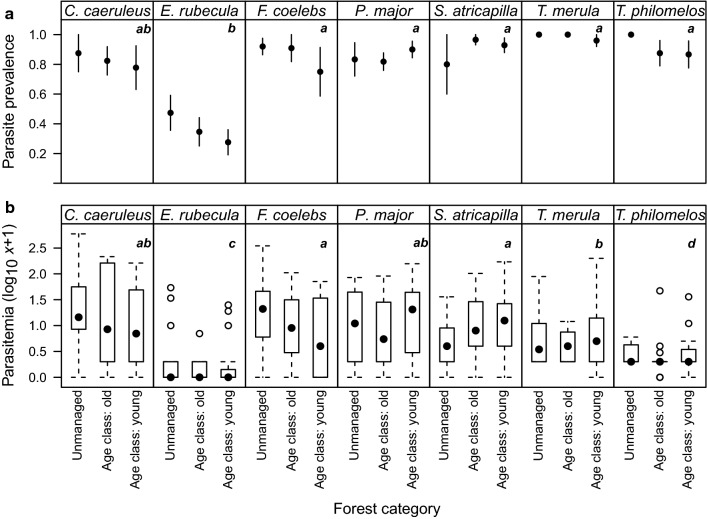
Mean parasite prevalence and median parasitemia of the seven avian host species. **a** Mean parasite prevalence (bars indicate 95% Confidence Intervals) and **b** median parasitemia of the seven avian host species within the beech forests of Schorfheide-Chorin, Germany, separated per forest category. Groups sharing a letter are not significantly different after Tukey post-hoc (Sample size: *C. caeruleus* n = 34, *E. rubecula* n = 74, *F. coelebs* n = 44, *P. major* n = 86, *S. atricapilla* n = 62, *T. merula* n = 59, *T. philomelos* n = 39)

*E. rubecula* had a significantly lower mean prevalence than the other avian species (35%; Table 1; Fig. 1a,). We did not find an effect of forest category on parasite prevalence for each avian species separately (Fig. 1a). Median parasitemia varied significantly between avian host species (Mood's median, χ2 = 85.68, df = 6, *P* < 0.01; Fig. 1b). We did not find significant differences in relation to parasitemia within an avian host species in relation to the forest categories (Fig. 1b). Our model selection approach did not show any effect of forest category on either parasite prevalence or parasitemia; instead, both asymmetry parameters were important factors to be included in a model explaining haemosporidian prevalence (Table 2). The analysis showed that tarsus asymmetry was negatively related to parasitemia and prevalence (Table 3, Additional file 1: Fig. S1, S2).

**Table 2:** Comparison of generalized linear mixed-effects models (GLMM) using a model selection approach

Response variable	Explanatory variables						
	Model description	k	AICc	ΔAICc	*wi*	Variance (fixed effects)	Variance (random effect)
a) Haemosporidian parasitemia	Primary 3 asymmetry + Tarsus asymmetry	5	588.65	0.00	0.92	0.012	0.255
	Primary 3 asymmetry + Tarsus asymmetry + Vector abundance (females)	6	593.99	5.34	0.06	0.015	0.250
	Primary 3 asymmetry + Tarsus asymmetry + Vector abundance (females) + H/L-ratio	7	596.56	7.91	0.02	0.018	0.243
	Primary 3 asymmetry + Tarsus asymmetry + Vector abundance (females) + H/L-ratio + Forest category	9	603.10	14.45	0.00	0.028	0.242
	Primary 3 asymmetry + Tarsus asymmetry + Vector abundance (females) + H/L-ratio + Forest category + Year	10	608.04	19.39	0.00	0.028	0.242
	Primary 3 asymmetry + Tarsus asymmetry + Vector abundance (females) + H/L-ratio + Forest category + Year + Leucocyte	11	613.57	24.93	0.00	0.028	0.245
	Primary 3 asymmetry	4	645.56	56.91	0.00	0.001	0.259
	Tarsus assymmetry	4	657.22	68.58	0.00	0.009	0.261
	Null	3	723.35	134.70	0.00	0.000	0.270
b) Haemosporidian prevalence	Primary 3 asymmetry + Tarsus asymmetry	4	273.99	0.00	0.70	0.010	0.182
	Primary 3 asymmetry + Tarsus asymmetry + Forest category	6	276.58	2.59	0.19	0.015	0.183
	Primary 3 asymmetry + Tarsus asymmetry + Forest category + Leucocytes	7	278.64	4.65	0.07	0.015	0.182
	Primary 3 asymmetry + Tarsus asymmetry + Forest category + Leucocytes + Vector abundance (females)	8	280.71	6.72	0.02	0.016	0.182
	Primary 3 asymmetry + Tarsus asymmetry + Forest category + Leucocytes + Vector abundance (females) + H/L-ratio	9	282.81	8.81	0.01	0.016	0.181
	Primary 3 asymmetry	3	293.59	19.60	0.00	0.000	0.176
	Tarsus asymmetry	3	299.53	25.53	0.00	0.013	0.189
	Tarsus asymmetry + Forest category + Leucocytes + Vector abundance (females) + H/L-ratio	8	307.70	33.71	0.00	0.021	0.193
	Null	2	323.55	49.56	0.00	0.000	na

Comparison of generalized linear mixed-effects models (GLMM) relating to habitat category, physical traits, prevalence and parasitemia of all haemosporidians, from birds sampled at Schorfheide, Germany in 2014 and 2015. The factor bird species was used as a random factor

**Table 3:** Model parameter estimates for predictors of haemosporidian parasitemia and haemosporidian prevalence

Response variable	Parameter	Level	Estimate	SE	p-value
Haemosporidian parasitemia	Intercept	NA	0.830	0.143	*< 0.001*
	Tarsus asymmetry	NA	− 12.173	5.802	*0.037*
	Primary 3 asymmetry	NA	14.072	12.944	0.278
Haemosporidian prevalence	Intercept	NA	2.359	0.580	*< 0.001*
	Tarsus asymmetry	NA	− 50.863	27.633	*0.066*
	Primary 3 asymmetry	NA	3.046	59.695	0.959

Model parameter estimates originate from the models with the lowest AICc. Significant *p*-values in italics and marginally significant in italics

We captured 4623 female vectors and the dataset included 15 species of Ceratopogonidae (biting midges), 12 species of Culicidae (mosquitoes) and 2 species of Simuliidae. The most observed species in terms of female abundance per vector family were: *Aedes cantans* (Culidiae), *Culicoides impunctatus* (Ceratopogonidae) and *Simulium lundstromi* (Simuliidae). The highest number of species for families Culicidae and Ceratopogonidae were observed in the old age forest category. We only caught black fly individuals in the age class young category (Additional file 1: Table S1). The model selection analysis showed that adding female vector abundance did not improve model fit in relation to parasite prevalence and parasitemia (Table 2).

**Fig. 2:**
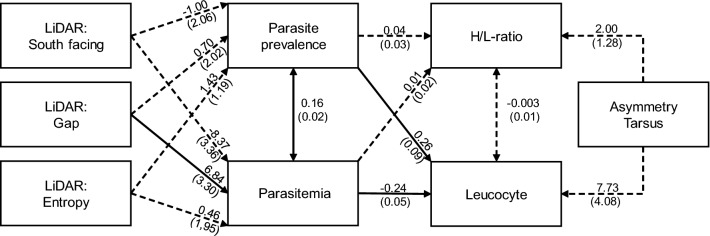
Path diagram of the best-fitted overall model for tarsus asymmetry (SEM). The number associated with each arrow is the parameter estimate. Thick solid lines are significant paths (*P* < 0.05), dashed lines are non-significant. The number indicates non-standardized parameter estimates; the number in parenthesis indicate the standard error. (Path diagrams of species-specific models, test statistics and full SEM results are detailed in Additional file 1: Tables S2–S5, Figs. S3, S4)

Accordingly, best-fitted SEMs (Structural Equation Models) indicated that female vector abundance has limited relevance in explaining parasite dynamics for our bird-dipteran-haemosporidian systems (Fig. 2; Additional file 1: Tables S2–S5). Incorporating the measure of vector abundance did not increase model fit, nor did model fit increase when we applied parasite genera and female vector specific relationships (i.e., Ceratopogonidae-*Haemoproteus*, Culicidae-*Plasmodium* and Simuliidae-*Leucocytozoon* (see the test statistics for the '1b' and '2b' models in Additional file 1: Tables S2–S5).

The overall SEM models, in which we analyzed all avian species together but with either primary 3 or tarsus asymmetry, conform to the idea that habitat management and structure (e.g., south facing slope, proportion of gaps) directly affect parasite infection probability and parasitemia first (i.e., top-down regulation; Fig. 2; Additional file 1: Fig. S2a).

However, there were differences in the outcome for each bird species. In the case of *P. major* and *T. merula*, forest structural variables first affected bird condition (i.e., bird asymmetries), which subsequently affected the probability of acquiring an infection as well as the severity of the infection (i.e., bottom-up regulation; Additional file 1: Tables S2–S5; Figs. S3, S4). In contrast, *S. atricapilla*, *E. rubecula* and *F. coelebs* were fitted better by the general pattern, where habitat structure directly affects parasite prevalence and parasitemia first (i.e., top-down regulation; Additional file 1: Tables S2–S5; Figs. S3, S4). Finally, in both overall SEM models, the correlation between parasite prevalence and parasitemia was significantly positive. (Fig. 2; Additional file 1: Tables S2–S5).

## Discussion

We analyzed how host-vector-parasite interactions respond to local forest management (GLMM) and forest structure (SEM). The general pattern indicated (i.e., the overall models) parasite regulation (i.e., top-down) from the perspective of the avian hosts. Habitat structure (e.g., south facing slope, proportion of gaps, entropy) first affects the probability of acquiring an infection (Fig. 2, Additional file 1: Figs. S3, S4), which then develops into a severe disease for the individual (measured by parasitemia), and subsequently forces birds to use limited resources to fight-off infections instead of using these resources for maintenance and physical development. For the birds, it implies a negative indirect effect on avian development, given that the birds would have to use resources to fight off infections (i.e., via immune responses; increased leucocyte numbers) instead of using them for growth and maintenance. We partially identified that the proportion of south facing forest (aspect) and the proportion of gaps affect avian haemosporidian transmission dynamics, but effects can vary across different bird species. Parasite transmission dynamics therefore occurred in different ways among different avian species (e.g. [18]). Importantly, vector abundance did not have an important effect on the transmission of avian haemosporidians in our study system [see also 20].

In one of our avian host species (*S. atricapilla*) both parasite prevalence and parasitemia were related to increased tarsus asymmetry, which might be an indirect effect of parasite infection. Haemosporidians are reported to affect tail feather growth [35, 36], and similarly other studies report increased asymmetry as a result of ectoparasite infections such as mites and cimicids, affecting the length of feathers, wings, and tarsus [24]. Body asymmetries can be a result of increased stress due to changes in habitat [37]. Asymmetries can be caused by an increased susceptibility and exposure to parasites [38] and negatively affect host fitness [39]. We found different infection dynamics for different bird species; for some species habitat structure seems to affect first their condition (bottom-up regulation), and for other species forest structure affects first the probability of an haemosporidian infection (top-down regulation). In order to tease apart if parasite infection leads to suboptimal bird conditions or vice versa, an experimental approach would be useful. Most likely, both scenarios, or mechanisms, occur in nature and will vary as a function of interacting species.

The method of parasite detection also played an important role; the total prevalence is higher when using a combination of both microscopy and molecular methods. Total parasite prevalence would therefore have been underestimated using only one method [40]. We also found differences in prevalence and parasitemia between avian species; thus, grouping parasite parameters over all avian host species would disregard subtle effects on actual infections. Our study confirms that parasite prevalence is variable across bird species [e.g. 18, 41]. Grouping host species may lead to a lower overall prevalence, such as when combining infections of 27 European species that lead to a mean prevalence of 26% [42]. Thus, the approach to better understand host-vector-parasite systems has to follow a reductionist framework, studying the same system across different geographical locations, gradually increasing the complexity (i.e., adding functional traits and other interacting organisms) in such a way that both contingent and general outcomes can be easily identified [43].

Contrary to our predictions, we did not find an effect of female vector abundance on haemosporidian parasitological parameters in any of our avian host species. We hypothesized that forest structure should indirectly affect parasite infection risk because of its known effects on vector abundance [16, 19]. Variation of parasite prevalence in birds has been observed as within-year seasonal variation, reflected by the seasonal variation of vector abundance, distribution or exposure [29]. To illustrate how the presence of vectors influences parasite dynamics, some studies have shown that parasites can be completely absent in specific sea bird populations or it can be as high as 80% in Afro-tropical water birds, which seems to be an effect of inhabiting either marine or freshwater environments (in combination with both phylogenetic and life-history traits) and possibly as a result of the abundance and presence of the Dipteran vectors [44–46]. However, our results suggest that vector abundance and seasonal variation are not always important drivers of infection risk [47]. Other factors and processes beyond vector abundance could be more influential, for example host-selection by the vector [48] or host-compatibility and specialization of the parasites [49]. Vectors of avian haemosporidians may play a major role in areas where they are extremely abundant [46], or when their influence is more pronounced at times when birds are prone to infections because of suppression of the immune system, e.g., after long distance migration [50] or during the breeding season [51]. Moreover, parasite induced phenotypic change in the host is a widespread phenomenon that also influences transmission dynamics. Some studies conducted on avian malaria have shown enhanced vector attraction toward infected hosts (indirect manipulation hypothesis; (e.g. [52]), implying a relation between infection status and vector behavior. Finally, our data is grouping the abundance of many different species within Diptera families, which can dilute subtle effects on transmission dynamics by particular vector species; thus, future studies should consider to tackle this issue whenever Diptera sample sizes of individual species allows (e.g. [53]).

We did not find any effect of the a priori determined forest categories on parasite infection status, which indicates limited effect of coarse forest management categories on infection dynamics in our system. However, in similar forest types and ecosystems, it was shown that parasite infection parameters of *F*. *coelebs* were significantly higher in beech forests compared to spruce and mixed forests, and for *S*. *atricapilla* there was significantly higher prevalence in spruce dominated forest stands than in mixed and beech forests [16, 19]. Such differences were interpreted as a result of increased intra-specific competition for suitable habitat (i.e., beech forest for *F. coelebs*), or as living in suboptimal habitats (i.e., *S. atricapilla* in spruce forests); in both cases there was a negative impact of forest structure parameters on body condition and immune status, increasing parasite infection risk [16, 19]. In our study, we analyzed host-vector-parasite interactions within beech forests only; beech forest age classes did not per se represent the intricate structural differences as a result of management, as for example spruce forest does. However, other studies show that parasite prevalence varies across bird species, even within similar habitat types [16, 18, 19]. In this study, using quantitative forest structural variables (i.e., LiDAR) allowed a better understanding of ecosystem processes or species distributions [54], and has value for disentangling the complex relationships between the effects of land use (management) and forest structure on host-parasite interactions [19]. We here confirm the relevance of fine-scale forest structural parameters (e.g., gaps, south facing slope, entropy) on ecosystem processes. Therefore, we recommend that future analyses of the effects of land use on host-vector-parasite systems include continuous structural parameters whenever possible, instead of relying on coarse habitat classes.

## Conclusions

We found that parasite prevalence and parasitemia in seven avian hosts are affected by differences in fine-scale habitat structural parameters. In some cases, fine-scale habitat translates into negative indirect impacts on bird condition (i.e., physical asymmetry). Land use, such as forest fragmentation, deforestation, and urbanization have large effects on parasite dynamics at local geographical scales (e.g. [25, 55]). Other studies have shown that land use management influences all components of the host-vector-parasite system: avian hosts [12], vector abundance (e.g. [28]), and parasite prevalence [20]. Our study confirms some parts of previous research but adding new insights and confirm that even subtle changes in forest structure, can have significant effects on host-vector-parasite interactions. Our study results have relevance to other vector-borne diseases and ecosystem processes, and it emphasizes the need to consider species-specific habitat effects on parasite dynamics for each host species.

## Methods

### Study area

Our study was part of the DFG Biodiversity Exploratories, a long-term and large-scale biodiversity research scheme in Germany [56]. The study site is located in the northeast of Germany, within the Schorfheide-Chorin Biosphere Reserve (52°58′ N, 13°45′ E). The Biosphere Reserve is a young lowland glacial landscape, covering about 1,300 km^2^, and altitude ranges between 3 and 140 m.a.s.l., with mean annual precipitation of 500–600 mm [56]. The study site contains 50 experimental forest plots, from which we sampled a total of 21 plots. All plots are 100 × 100 m with at least a 30 m buffer of the same forest structure per plot and within the buffer. We focused on forest stands dominated (i.e., > 70% canopy cover) by European beech (*Fagus sylvatica*) and selected three forest categories based on median stand age. The three forest categories were: young (age class; < 136 years), old (age class; > 136 years) and unmanaged (for at least 60 years). We selected seven replicate forest plots for each of the three forest categories, resulting in 21 plots (Additional file 1: Table S6).

We further categorized forest structure and heterogeneity at the plot level using LiDAR (Light Detection and Ranging) to derive continuous forest structural parameters [57]. LiDAR is an active remote sensing system that can be used to map structure including vegetation height, density and other characteristics over large regions (e.g. ecosystem or regional scale). LiDAR variables were selected based on their relevance to the research topic of bird-vector-parasite interactions: forests entropy (local vertical variation in canopy structure or canopy surface heterogeneity), gap (proportion of canopy characterized as gap), and south facing aspect (proportion of south facing canopy) [c.f. 19, 20, 58].

### Insect vectors

We captured potential vectors on the same day when we captured avian hosts. We used one BG Sentinel Trap per forest plot, baited with CO_2_ (outflow of 200 g per day), Biogents Sweet-scent and an ultraviolet module to improve catch rate (Biogents AG, Regensburg, Germany), traps were placed in the center of the forest plot on the forest floor. All traps ran for 18 h, starting typically around 1 p.m. until the next morning. Insects were placed in a freezer at − 20 °C before immersion in 80% analytical grade ethanol. Insects were classified to the family level [59] and sorted to sex by morphology using a stereo microscope, species were determined by PCR and sequencing [20, 60]. Vector abundance was calculated as the sum of all female individuals per forest plot for the families Culicidae, Ceratopogonidae, and Simuliidae that respectively transmit avian haemosporidians of the genera *Plasmodium*, *Haemoproteus* and *Leucocytozoon* [21].

### Host model species

Seven avian host species were selected for our study based on capture events and their habitat preferences: Eurasian blue tit (*Cyanistes caeruleus*), European robin (*Erithacus rubecula*), common chaffinch (*Fringilla coelebs*), great tit (*Parus major*), Eurasian blackcap (*Sylvia atricapilla*), common blackbird (*Turdus merula*) and song thrush (*Turdus philomelos*). We merged all captures of a given avian host species within each forest category.

All seven avian host species are common in forests, but with varying preferences for forest structure and forest types. *C. caeruleus* prefers all kinds of tree species, avoiding conifers and open stands [61]. *E. rubecula* prefers medium dense forests, both broad leaved and mixed woodlands with cool and moist places while avoiding open spaces [62]. *F. coelebs* is an arboreal species with a preference for mixed deciduous forests during the breeding season but can be found also in other mixed and conifer forests [63]. *P. major* is a lowland species, avoiding conifer forest stands, and prefers mixed deciduous over pure deciduous forest stands, also prefers high structure of the undergrowth for cover and foraging [61]. *S. atricapilla* forages and sings higher in the canopy, requires not too dense undergrowth for nesting and particularly avoids pure conifer forest stands [64]. Both *T. merula* and *T. philomelos* tolerate wet and humid conditions, especially for invertebrate food items, and both can be found in diverse habitats, from dense woodlands to open landscape. *T. merula* is least frequent in the canopy and forages in all other layers and has no preference for conifer versus broadleaved forest stands. *T. philomelos* can be found where tree species provide enough shade and undergrowth, like beech forests [62].

### Bird sampling and blood smear preparation

Birds were sampled three times in 2014 and two times in 2015 on each plot from April until June—with at least 14 days in-between sampling visits of the same plot [65]. We captured birds from April until June using eight mist nets of 9 m × 2.5 m (mesh size 16 mm, nylon, Ecotone), placed at four locations within each experimental plot. Nets were opened 30 min after sunrise and left open for five consecutive hours aiming at the bird activity peak. Nets were checked with intervals of approximately 20 min. We used playback stations near the nets, playing territorial songs of the seven avian host species for improved capture success. We took measurements on both sides of each individual using a digital caliper (tarsus and third primary feather from the outside, i.e., primary 3) [66]. Asymmetry was calculated as a ratio between right and left measurement, and as an absolute deviation from 1 (a ratio of 1 implies symmetry) to avoid using a measure for directed asymmetry. Birds were ringed and any recaptures within the same sampling period were excluded from the analysis.

About 30 ɥl of blood were taken from the brachial vein using a micro-capillary tube. Blood smears were prepared shortly after drawing the blood and another part was saved for molecular diagnostics [21]. Blood smears were air dried, fixed in absolute methanol and stained with Giemsa for microscopic analyses (a solution consisting of 1.0 g Na_2_HPO_4_ + 0.7 g KH_2_PO_4_ + 1 L of double distilled H_2_O).

### Hematological parameters (parasite prevalence, parasitemia, leucocyte count and H/L-ratio)

Blood smears were scanned with a Leica microscope (DM 5500B) and analyzed in oil immersion at 1000× magnification. Images of blood slides were screened manually to count the number of blood parasites (per 10,000 erythrocytes) as a measure of parasite load (i.e., parasitemia). We counted the leucocytes and determined heterophil to lymphocyte ratio (H/L-ratio) to assess birds' immune response [67]. The H/L-ratio is a measure of stress in birds and is positively related to the magnitude of the stressor [68]. We used a nested PCR protocol which allows simultaneous screening of Haemosporidia of the genera *Plasmodium*, *Haemoproteus* and *Leucocytozoon* [69], but cannot make a distinction between the first two genera.

For the analysis, we used a combined prevalence measure based on both PCR and microscopy. This means that when either technique showed a positive infection, we regarded this avian host as infected. If we detected an infection only through PCR, we assumed a very low parasitemia (a parasite count of 1) not detected by using the microscope [70]. However, in such cases we repeated each PCR at least two times to avoid false positives.

### Data analysis and statistics

All data was log-transformed (log_10_ *x* + 1) except for parasite prevalence (binomial), and were analyzed using R, version 4.0.1 [71]. Mood's median tests were carried out if the data did not follow normality (i.e., with parasitemia) using the R-package "RVAideMemoire" [72] using the approach as outlined in Rózsa et al*.* [73, 74]. Generalized Linear Mixed Models (GLMM) were applied using the "lme4" package [75] and test results were obtained using the "lmerTest" and "piecewiseSEM" packages [76, 77], the factor bird species was used as a random effect in order to capture species-specific responses [18, 19]. Results from tests were considered statistically significant if the *P*-value was less than 0.05.

Initially, we tested the effect of forest categories on parasite prevalence or parasitemia per avian host species. The effect of forest category (factor, 3-levels) on parasite prevalence was tested by fitting one GLM (binomial data structure) and Mood's median tests for the effect on parasitemia (data not normally distributed). Subsequently, we used a model selection approach in combination with Generalized Linear Mixed Models (GLMM) to analyze the data in a broader context to understand mean associations between parasite infections and forest categories, avian host species, vector abundance, body asymmetry and immune response. We tested the effect of forest category, female vector abundance, H/L-ratio, leucocyte count and both tarsus and primary-3 asymmetry on both parasite prevalence and parasitemia. Except for 'forest category', all predictors were continuous. We stepwise removed one predictor variable at a time (based on the highest *P*-value) and selected a best-fitted model based on the largest difference of the Akaike Information Criterion corrected for small sample sizes (ΔAICc) to the full model, considering a better model-fit with decreasing AICc from the initial model [78, 79]. The results of the model selection formed the basis of the subsequent approach were model estimates were acquired using R-package "MuMIn" [80]

In order to have a more thorough understanding of the ecological dynamics in our host-vector-parasite systems, we applied Structural Equation Modeling (SEM), which allows analyzing alternative pathways of associations among variables at the same time, helping to determine the strength of association (covariance) between variables [81, 82]. We tested the original models, as well as the amended models that included a variable for vector abundance. In the model selection approach, we grouped parasite genera and vector families together, but since the parasite genera of *Plasmodium*, *Haemoproteus* and *Leucocytozoon* are each transmitted by dipteran vectors of the different dipteran families (i.e., *Plasmodium*-Culicidae, *Haemoproteus*-Ceratopogonidae and *Leucocytozoon*-Simuliidae), we made further additional parasite-vector specific analysis. However, *Plasmodium* and *Haemoproteus* infections cannot be separated with the PCR protocol of Hellgren et al. [69] and thus their respective vectors were grouped [82]. We included parasite genus and vector family explicitly in the models (*Plasmodium*-Culicidae and *Haemoproteus*-Ceratopogonidae vector abundance). We applied all models to all bird species merged and separately for each bird species (i.e., overall and species-specific model; Additional file 1: Figs. S2, S3; Tables S2–S5) and separated the models by each tarsus and primary 3 asymmetries. Each model was analyzed separately with either the full dataset, or with sub-selections in case of the species-specific models. Models with a *P*-value (based on the chi-square value, χ^2^) < 0.05 were rejected, and we subsequently retained models with both Goodness-of-Fit Index (GFI) and Comparative Fit Index (CFI) of 0.9 or higher [83]. From all models that met the criteria, the model with the lowest AIC (Akaike Information Criterion) value was selected as the best-fitted model [79]. In the figures, path coefficients are indicated as non-standardized parameter estimates and the value in parenthesis indicate standard error values [83, 84]. SEMs were created using the R-package "lavaan", version 0.5–23.1097 [84].

## Supplementary information

**Additional file 1:** Additional figures and tables related to the statistical analyses.

**Additional file 2:** Raw data.

## Data Availability

All data generated or analyzed during this study are included in this published article and its Additional files.

## Abbreviations

GLM: generalized linear model
GLMM: generalized linear mixed model
H/L-ratio: heterophil to lymphocyte ratio
LiDAR: light detection and ranging
PCR: polymerase chain reaction
SEM: structural equation model
