# Supplementary material for: Effects of forest structure on the interaction between avian hosts, dipteran vectors and haemosporidian parasites
Source: BMC Ecol. 2020 Aug 19;20:47. doi: 10.1186/s12898-020-00315-5 (PMC7437053; doi:10.1186/s12898-020-00315-5)
Supplement: Supplementary file 1 — Additional file 1: Additional figures and tables related to the statistical analyses. [file 12898_2020_315_MOESM1_ESM.docx]

# Additional file 1

## Additional file 1: Figures

| **Fig. S1a-b** | Visualization of the effect of (A) primary asymmetry and (B) tarsus asymmetry on parasitemia separated per bird species. |
| --- | --- |
| **Fig. S2a-b** | Visualization of the effect of (A) primary asymmetry and (B) tarsus asymmetry on parasite prevalence separated per bird species. |
| **Fig. S3a-c** | Path diagrams of the best fitted species-specific structural equation models (SEM) for tarsus asymmetry. |
| **Fig. S4a-f** | Path diagrams of the best fitted species-specific structural equation models (SEM) for primary asymmetry. |

## Additional file 1: Tables

| **Table S1** | Abundance of observed vector species per forest category within the study site |
| --- | --- |
| **Table S2** | Test statistics for all Structural Equation Models (SEM) for tarsus asymmetry |
| **Table S3** | Test statistics for all Structural Equation Models (SEM) for primary 3 asymmetry |
| **Table S4** | Summary of the best-fitted Structural Equation Models (SEM) |
| **Table S5** | Detailed results for the best fitted Structural Equation Models (SEM) |
| **Table S6** | Overview of the sampled plots regarding protection status |

## Additional file 1: References cited in Additional file 1

# Additional file 1: Figures

**Figure S1.** Visualization of the effect of (a) primary asymmetry and (b) tarsus asymmetry on parasitemia separated per bird species. According to the model selection approach (GLMM), the trend is generally positive for primary asymmetry, while for tarsus asymmetry this is negative.
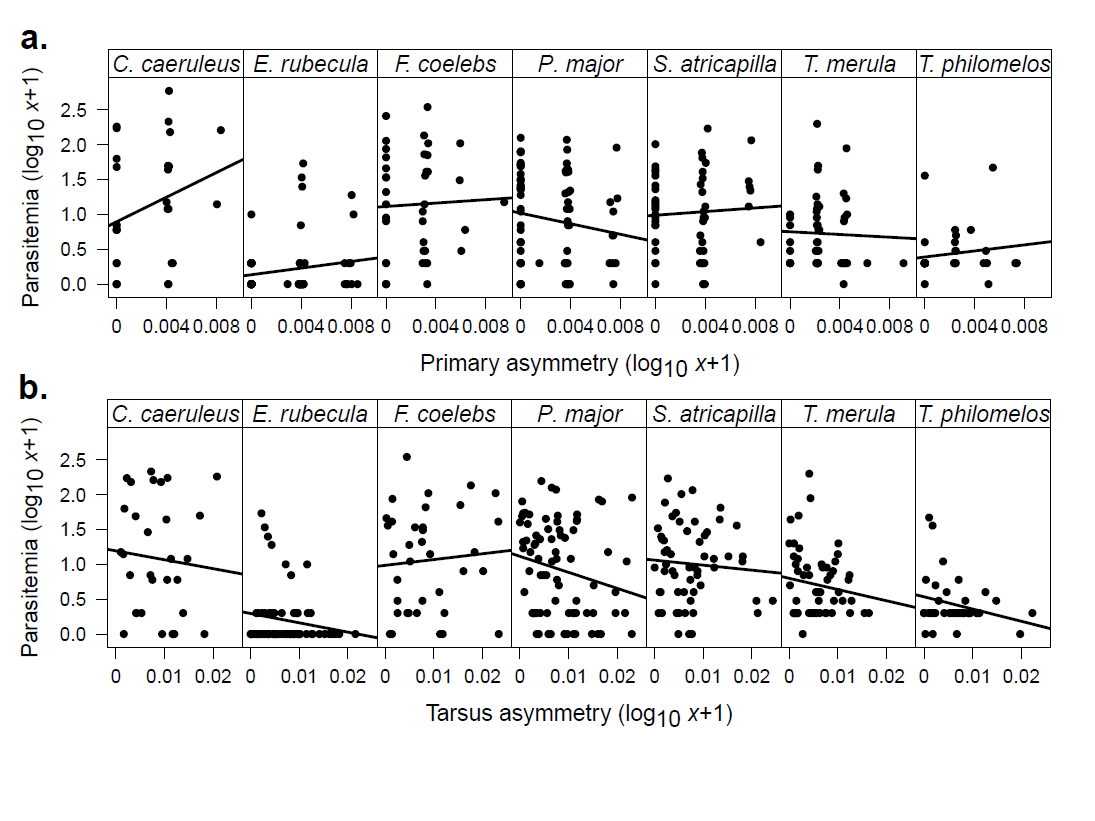


**Figure S2.** Visualization of the effect of (a) primary asymmetry and (b) tarsus asymmetry on parasite prevalence separated per bird species. According to the model selection approach (GLMM), the trend is generally positive for primary asymmetry, while for tarsus asymmetry this is negative.


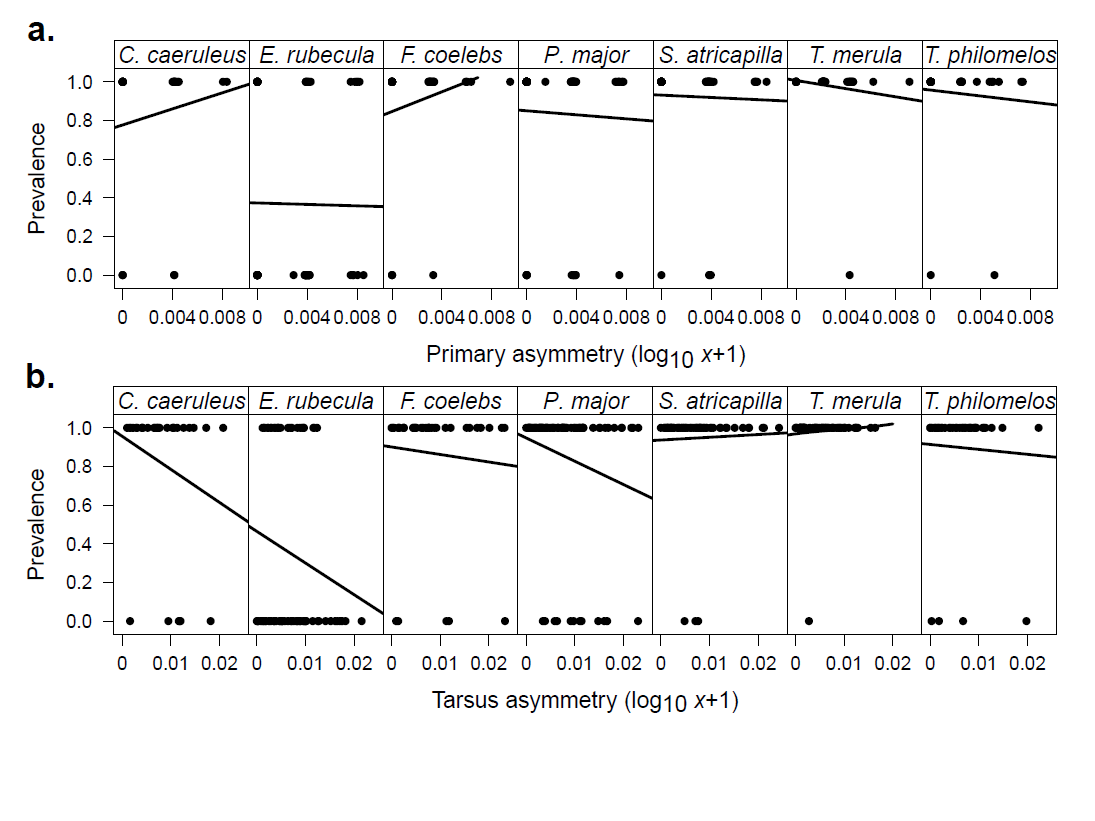


**Figure S3a-c.** Path diagrams of the best fitted species-specific structural equation models (SEM) for tarsus asymmetry. The number associated with each arrow is the parameter estimate, in brackets is indicated the standard error (H/L-ratio; heterophil to lymphocyte ratio). Solid lines are significant paths (*P* < 0.05) and dashed lines are nonsignificant. Test statistics of the SEMs are found in Table S5. Species-specific models for (a) *P. major*, (b) *S. atricapilla*, and (c) *T. merula*. Full SEM results are found in Supplemental Information Table S2, S4, S5.

(a)


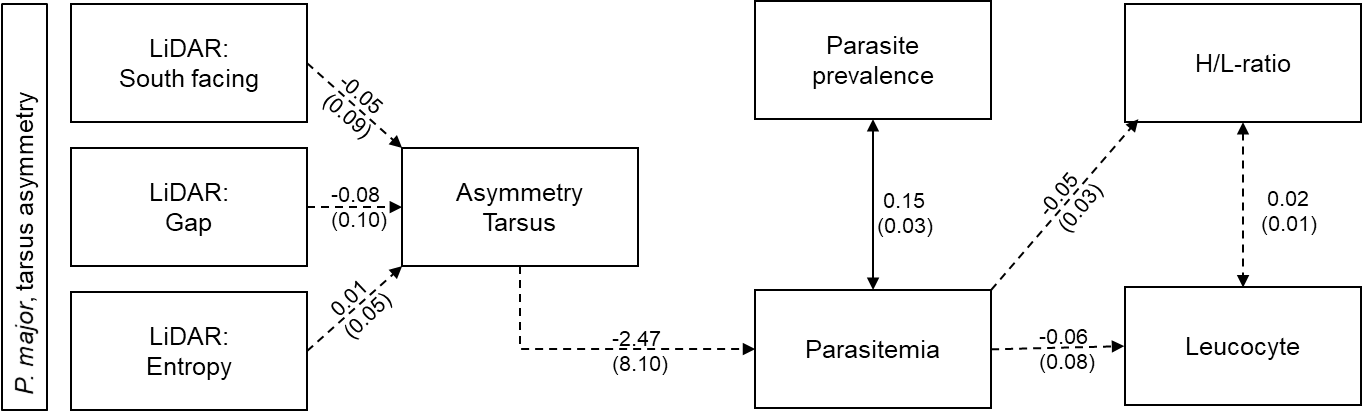


(b)


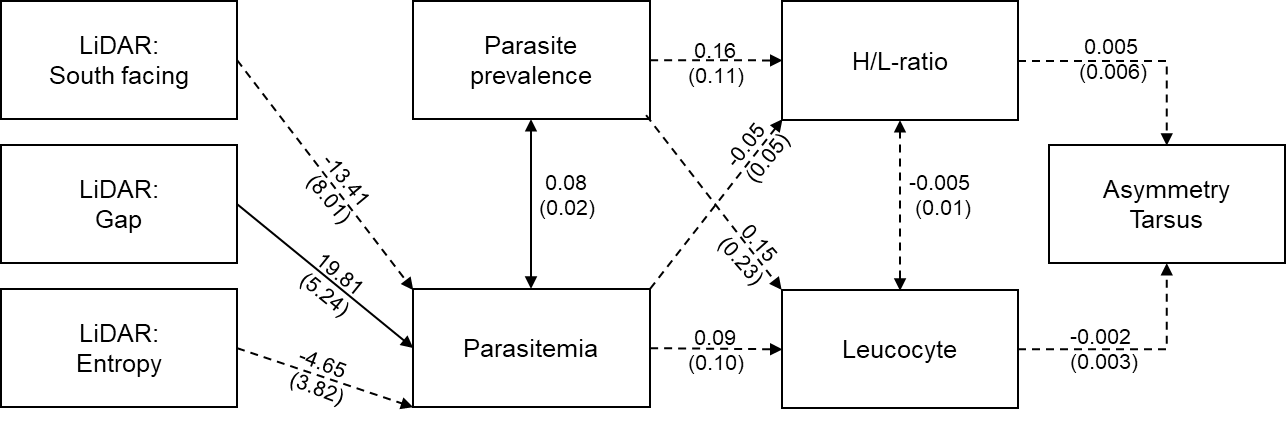


(c)


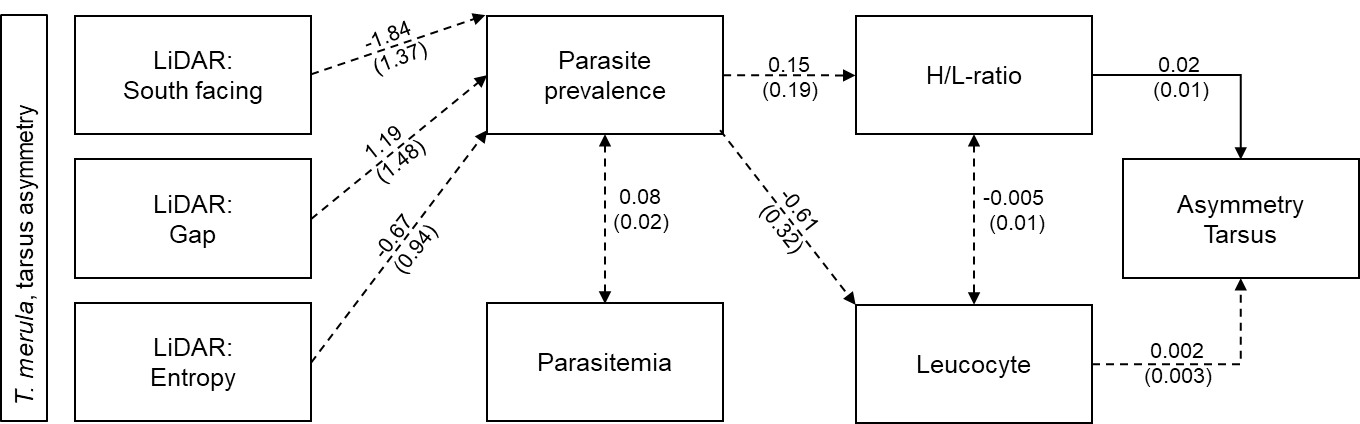


**Figure S4a-f.** Path diagrams of the best fitted structural equation models (SEM) for primary 3 asymmetry. The number associated with each arrow is the parameter estimate, in brackets is indicated the standard error (H/L-ratio; heterophil to lymphocyte ratio). Solid lines are significant paths (*P* < 0.05) and dashed lines are nonsignificant. Test statistics of the SEMs are found in Table S5. Species-specific models for (a) overall, (b) *E. rubecula*, (c) *F. coelebs*, (d) *P. major*, (e) *S. atricapilla* and (f) *T. merula*. Full SEM results are found in Supplemental Information Table S3, S4, S5.

(a)


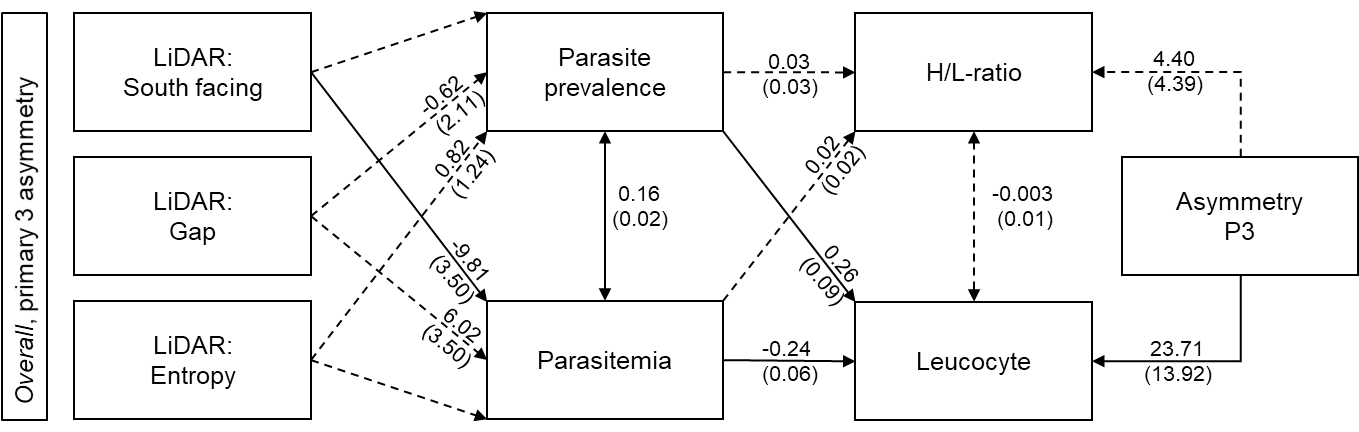


(b)


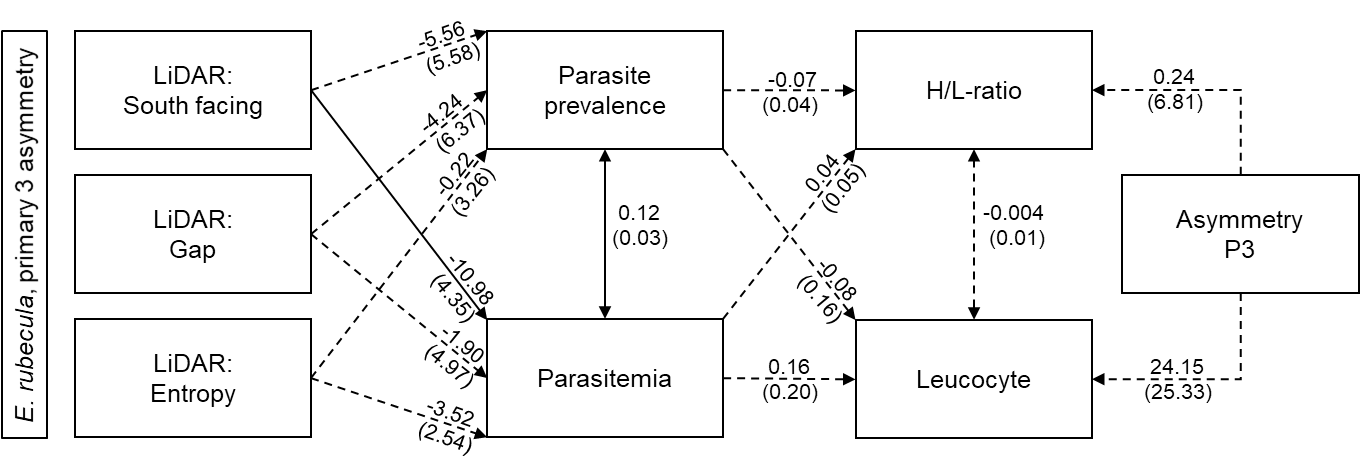


(c)


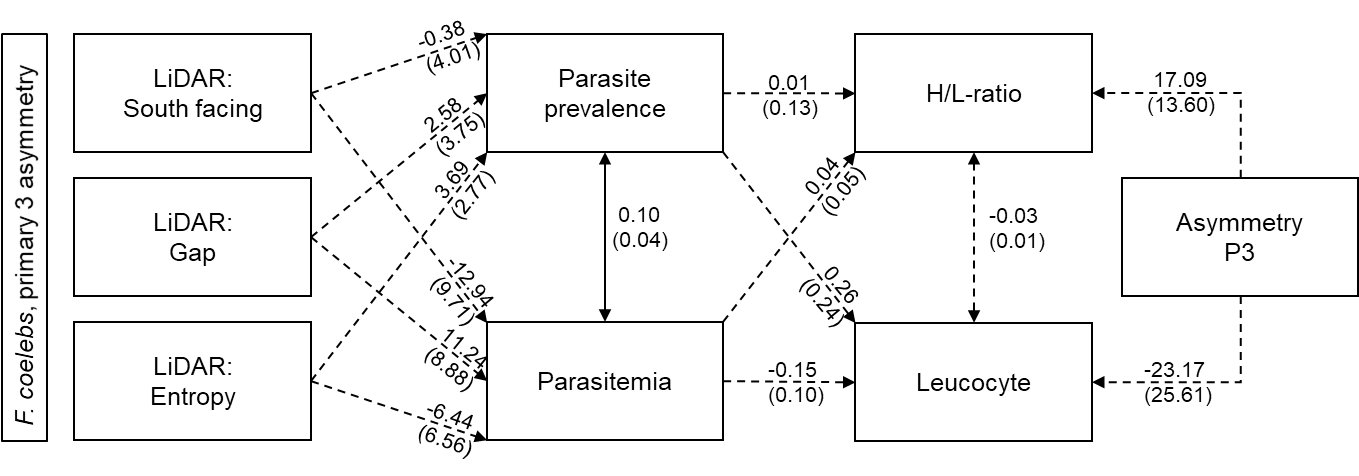


(d)


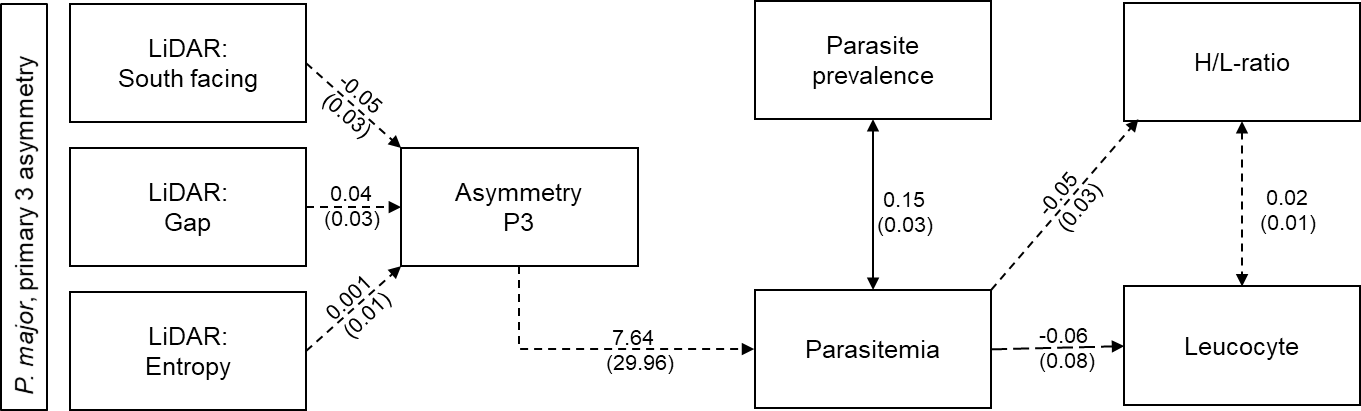


(e)


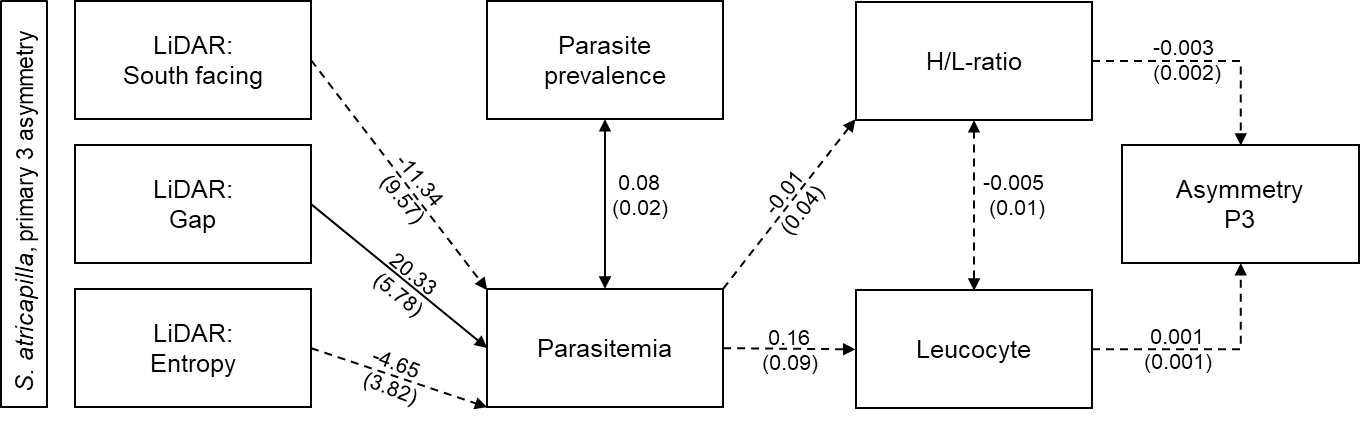


(f)


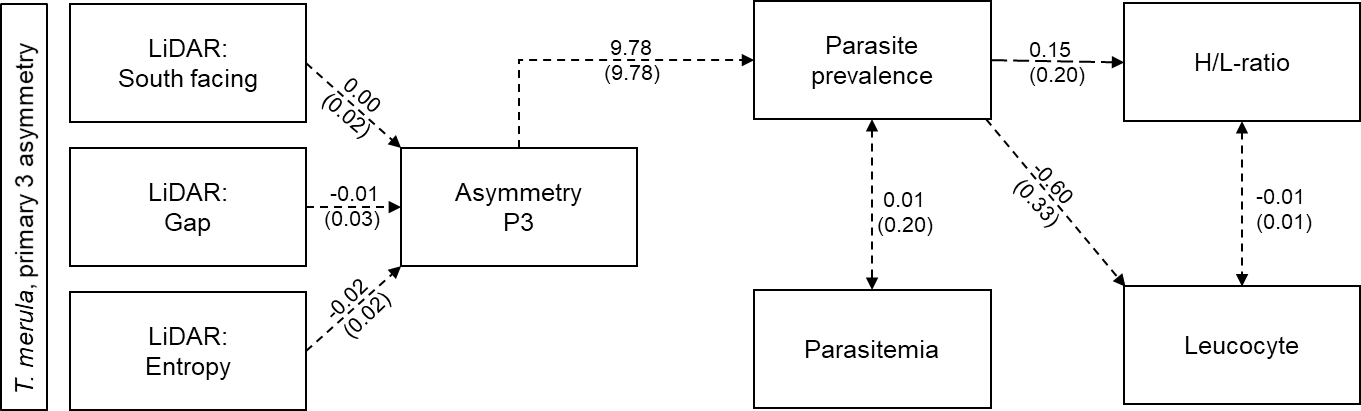


# Additional file 1: Tables

**Table S1.** Abundance of observed vector species per forest category within the study site (Schorfheide-Chorin Biosphere Reserve in Northeastern Germany). Shown are numbers of female individuals. (The list is adapted from W van Hoesel, A Marzal, S Magallanes, D Santiago-Alarcon, S Ibáñez-Bernal and SC Renner [1]).

|  |  | **Forest category** |  |  |
| --- | --- | --- | --- | --- |
| **Diptera family** | **Species** | **Unmanaged** | **Age class: old** | **Age class: young** |
| Culidae | *Aedes annulipes* | 6 | 7 | 236 |
|  | *Aedes cantans* | 219 | 3 | 319 |
|  | *Aedes cataphylla* |  | 1 |  |
|  | *Aedes cinereus* | 19 | 5 | 3 |
|  | *Aedes communis* |  | 12 | 49 |
|  | *Aedes diantaeus* |  | 121 |  |
|  | *Aedes leucomelas* |  |  | 1 |
|  | *Aedes punctor* | 53 | 4 | 6 |
|  | *Aedes riparius* | 1 |  |  |
|  | *Anopheles claviger* | 2 | 2 | 3 |
|  | *Anopheles plumbeus* | 7 | 7 | 1 |
|  | *Coquillettidia richiardii* | 1 | 2 |  |
| Ceratopogonidae | *Culicoides circumscriptus* |  |  | 1 |
|  | *Culicoides clastrieri* | 49 | 15 |  |
|  | *Culicoides duddingstoni* |  | 3 |  |
|  | *Culicoides festivipennis* | 263 | 409 | 484 |
|  | *Culicoides impunctatus* | 312 | 577 | 416 |
|  | *Culicoides kibunensis* | 15 | 68 |  |
|  | *Culicoides lupicaris* |  | 27 |  |
|  | *Culicoides newsteadi* | 88 | 20 | 14 |
|  | *Culicoides obsoletus* |  |  | 7 |
|  | *Culicoides pictipennis* | 144 | 160 | 108 |
|  | *Culicoides poperinghensis* |  | 17 |  |
|  | *Culicoides punctatus* | 240 | 26 |  |
|  | *Culicoides scoticus* |  | 13 | 4 |
|  | *Culicoides segnis* |  |  | 9 |
|  | *Culicoides truncorum* | 48 |  |  |
| Simuliidae | *Simulium lundstromi* |  |  | 4 |
|  | *Simulium noelleri* |  |  | 1 |
| Total Culicidae |  | 308 | 164 | 618 |
| Total Ceratopogonidae |  | 1159 | 1335 | 1043 |
| Total Simuliidae |  | 0 | 0 | 5 |

**Table S2.** Test statistics for all models for tarsus asymmetry (SEM). Statistics that meet the criteria are indicated in gray shading (P > 0.05; GFI > 0.9; CFI > 0.9). The best-fitted model for each species is marked in grey. ΔAIC only given when all criteria were met. For the explanation of the model names, see footnote.

| *Tarsus Asymmetry: all bird species* | | | | | | | | |
| --- | --- | --- | --- | --- | --- | --- | --- | --- |
| **Model name** | **n** | **dF** | **Chisq** | **P-value** | **GFI** | **CFI** | **AIC** | **ΔAIC** |
| 1a7 | 387 | 8 | 4.751 | 0.784 | 0.994 | 1.000 | -7889.141 | 0.000 |
| 1a1 | 387 | 11 | 7.213 | 0.782 | 0.993 | 1.000 | -7884.679 | 4.462 |
| 1a5 | 387 | 16 | 19.569 | 0.240 | 0.980 | 0.980 | -7882.323 | 6.818 |
| 2a1 | 387 | 14 | 19.646 | 0.142 | 0.980 | 0.969 | -7878.246 | 10.895 |
| 1a6 | 387 | 14 | 10.006 | 0.762 | 0.994 | 1.000 | -7875.886 | 13.255 |
| 2a5 | 387 | 17 | 30.205 | 0.025 | 0.971 | 0.927 | -7873.686 |  |
| 1a4 | 387 | 14 | 14.472 | 0.415 | 0.991 | 0.997 | -7871.420 | 17.721 |
| 2a2 | 387 | 12 | 13.241 | 0.352 | 0.992 | 0.993 | -7868.650 | 20.490 |
| 1a3 | 387 | 16 | 33.447 | 0.006 | 0.968 | 0.904 | -7868.445 |  |
| 2a4 | 387 | 17 | 38.680 | 0.002 | 0.963 | 0.880 | -7865.211 |  |
| 1a2 | 387 | 13 | 167.337 | 0.000 | 0.876 | 0.148 | -7728.555 |  |
| 2a3 | 387 | 15 | 179.769 | 0.000 | 0.866 | 0.091 | -7720.122 |  |
| 1b7 | 387 | 17 | 27.709 | 0.048 | 0.972 | 0.945 | -6955.213 |  |
| 1b11 | 387 | 11 | 17.480 | 0.094 | 0.982 | 0.967 | -6953.443 | 935.698 |
| 1b1 | 387 | 20 | 30.171 | 0.067 | 0.975 | 0.948 | -6950.751 | 938.390 |
| 1b8 | 387 | 14 | 19.942 | 0.132 | 0.983 | 0.969 | -6948.981 | 940.160 |
| 1b5 | 387 | 23 | 39.888 | 0.016 | 0.967 | 0.913 | -6947.034 |  |
| 2b2 | 387 | 15 | 25.439 | 0.044 | 0.979 | 0.946 | -6945.484 |  |
| 2b1 | 387 | 17 | 31.675 | 0.017 | 0.974 | 0.925 | -6943.247 |  |
| 1b10 | 387 | 18 | 22.927 | 0.193 | 0.987 | 0.975 | -6941.995 | 947.146 |
| 2b5 | 387 | 21 | 42.318 | 0.004 | 0.966 | 0.890 | -6940.605 |  |
| 1b6 | 387 | 21 | 30.325 | 0.086 | 0.983 | 0.952 | -6940.598 | 948.543 |
| 1b4 | 387 | 21 | 31.235 | 0.070 | 0.983 | 0.947 | -6939.687 | 949.454 |
| 1b9 | 387 | 18 | 27.798 | 0.065 | 0.985 | 0.950 | -6937.124 | 952.017 |
| 1b3 | 387 | 23 | 50.210 | 0.001 | 0.961 | 0.860 | -6936.712 |  |
| 2b4 | 387 | 21 | 51.840 | 0.000 | 0.959 | 0.841 | -6931.082 |  |
| 1b2 | 387 | 22 | 189.498 | 0.000 | 0.880 | 0.139 | -6795.425 |  |
| 2b3 | 387 | 18 | 191.882 | 0.000 | 0.878 | 0.106 | -6785.041 |  |
| 1b7x | 384 | 17 | 29.554 | 0.030 | 0.970 | 0.895 | -6684.471 |  |
| 1b8x | 384 | 14 | 19.311 | 0.153 | 0.984 | 0.955 | -6680.714 | 1208.427 |
| 1b1x | 384 | 20 | 31.499 | 0.049 | 0.974 | 0.902 | -6680.526 | 1208.615 |
| 2b2x | 384 | 15 | 29.132 | 0.015 | 0.976 | 0.880 | -6672.894 |  |
| 2b1x | 384 | 17 | 34.169 | 0.008 | 0.972 | 0.854 | -6671.857 |  |
| 1b3x | 384 | 23 | 47.448 | 0.002 | 0.962 | 0.792 | -6670.577 |  |
| 1b5x | 384 | 23 | 49.199 | 0.001 | 0.960 | 0.777 | -6668.826 |  |
| 1b9x | 384 | 18 | 27.589 | 0.069 | 0.985 | 0.918 | -6668.436 | 1220.705 |
| 1b10x | 384 | 18 | 31.634 | 0.024 | 0.983 | 0.884 | -6664.391 |  |
| 2b4x | 384 | 21 | 50.217 | 0.000 | 0.960 | 0.751 | -6663.808 |  |
| 1b6x | 384 | 21 | 39.545 | 0.008 | 0.978 | 0.842 | -6662.480 |  |
| 2b5x | 384 | 21 | 52.426 | 0.000 | 0.958 | 0.733 | -6661.599 |  |
| 1b2x | 384 | 22 | 104.555 | 0.000 | 0.923 | 0.298 | -6611.470 |  |
| 2b3x | 384 | 18 | 107.204 | 0.000 | 0.921 | 0.241 | -6600.821 |  |
| 1b4x | 384 | 30 | 274.840 | 0.000 | 0.899 | 0.442 | -6357.599 |  |
| 1b11x | 384 | 14 | 5372.810 | 0.000 | 0.712 | 0.022 | -5778.415 |  |
| *Tarsus Asymmetry: Cyanistes caeruleus* | | | | | | | | |
| **Model name** | **n** | **dF** | **Chisq** | **P-value** | **GFI** | **CFI** | **AIC** | **ΔAIC** |
| 1a5 | 33 | 16 | 18.377 | 0.302 | 0.843 | 0.908 | -712.134 |  |
| 1a3 | 33 | 16 | 22.029 | 0.142 | 0.810 | 0.767 | -708.481 |  |
| 1a7 | 33 | 8 | 15.690 | 0.047 | 0.836 | 0.726 | -706.821 |  |
| 2a5 | 33 | 17 | 26.167 | 0.071 | 0.788 | 0.645 | -706.344 |  |
| 2a4 | 33 | 17 | 27.311 | 0.054 | 0.772 | 0.601 | -705.200 |  |
| 1a1 | 33 | 11 | 16.464 | 0.125 | 0.857 | 0.788 | -704.047 |  |
| 2a1 | 33 | 14 | 26.004 | 0.026 | 0.789 | 0.535 | -700.506 |  |
| 1a6 | 33 | 14 | 18.344 | 0.192 | 0.896 | 0.832 | -696.167 |  |
| 1a4 | 33 | 14 | 20.736 | 0.109 | 0.883 | 0.739 | -693.775 |  |
| 2a2 | 33 | 12 | 19.963 | 0.068 | 0.888 | 0.692 | -690.548 |  |
| 1a2 | 33 | 13 | 36.900 | 0.000 | 0.703 | 0.075 | -687.610 |  |
| 2a3 | 33 | 15 | 43.496 | 0.000 | 0.694 | 0.000 | -685.015 |  |
| 1b3 | 33 | 23 | 31.553 | 0.110 | 0.787 | 0.762 | -638.221 |  |
| 1b5 | 33 | 23 | 32.081 | 0.098 | 0.790 | 0.747 | -637.693 |  |
| 1b7 | 33 | 17 | 28.918 | 0.035 | 0.774 | 0.688 | -636.856 |  |
| 1b1 | 33 | 20 | 29.691 | 0.075 | 0.800 | 0.730 | -634.082 |  |
| 2b4 | 33 | 21 | 34.180 | 0.035 | 0.761 | 0.633 | -631.593 |  |
| 2b5 | 33 | 21 | 35.169 | 0.027 | 0.765 | 0.606 | -630.604 |  |
| 1b11 | 33 | 11 | 23.219 | 0.016 | 0.811 | 0.680 | -630.555 |  |
| 1b3x | 33 | 23 | 26.843 | 0.263 | 0.805 | 0.874 | -628.073 |  |
| 1b8 | 33 | 14 | 23.992 | 0.046 | 0.832 | 0.722 | -627.781 |  |
| 1b7x | 33 | 17 | 24.001 | 0.119 | 0.804 | 0.786 | -626.914 |  |
| 2b2 | 33 | 15 | 27.742 | 0.023 | 0.811 | 0.645 | -626.031 |  |
| 2b1 | 33 | 17 | 32.236 | 0.014 | 0.775 | 0.576 | -625.538 |  |
| 1b1x | 33 | 20 | 24.163 | 0.235 | 0.829 | 0.864 | -624.753 |  |
| 1b4 | 33 | 21 | 30.259 | 0.087 | 0.855 | 0.742 | -623.515 |  |
| 1b6 | 33 | 21 | 32.047 | 0.058 | 0.849 | 0.693 | -621.726 |  |
| 2b4x | 33 | 21 | 31.261 | 0.069 | 0.776 | 0.664 | -619.654 |  |
| 1b9 | 33 | 18 | 28.550 | 0.054 | 0.861 | 0.706 | -619.224 |  |
| 1b10 | 33 | 18 | 29.061 | 0.048 | 0.862 | 0.692 | -618.713 |  |
| 1b8x | 33 | 14 | 19.384 | 0.151 | 0.860 | 0.824 | -617.531 |  |
| 2b2x | 33 | 15 | 22.595 | 0.093 | 0.840 | 0.752 | -616.320 |  |
| 1b5x | 33 | 23 | 39.181 | 0.019 | 0.748 | 0.471 | -615.734 |  |
| 2b1x | 33 | 17 | 28.497 | 0.039 | 0.800 | 0.624 | -614.418 |  |
| 1b2x | 33 | 22 | 38.668 | 0.015 | 0.718 | 0.455 | -614.247 |  |
| 1b2 | 33 | 22 | 56.073 | 0.000 | 0.677 | 0.052 | -611.700 |  |
| 1b9x | 33 | 18 | 23.942 | 0.157 | 0.881 | 0.806 | -608.973 |  |
| 2b5x | 33 | 21 | 43.557 | 0.003 | 0.718 | 0.262 | -607.359 |  |
| 2b3x | 33 | 18 | 38.540 | 0.003 | 0.737 | 0.328 | -606.375 |  |
| 2b3 | 33 | 18 | 56.031 | 0.000 | 0.681 | 0.000 | -603.743 |  |
| 1b6x | 33 | 21 | 37.335 | 0.015 | 0.831 | 0.466 | -601.581 |  |
| 1b10x | 33 | 18 | 35.564 | 0.008 | 0.837 | 0.426 | -597.351 |  |
| 1b4x | 33 | 30 | 75.689 | 0.000 | 0.762 | 0.427 | -588.638 |  |
| 1b11x | 33 | 14 | 349.252 | 0.000 | 0.691 | 0.071 | -548.295 |  |
| *Tarsus Asymmetry: Erithacus rubecula* | | | | | | | | |
| **Model name** | **n** | **dF** | **Chisq** | **P-value** | **GFI** | **CFI** | **AIC** | **ΔAIC** |
| 2a4 | 71 | 17 | 25.953 | 0.075 | 0.876 | 0.849 | -1635.790 |  |
| 1a5 | 71 | 16 | 24.214 | 0.085 | 0.888 | 0.862 | -1635.529 |  |
| 1a7 | 71 | 8 | 16.983 | 0.030 | 0.899 | 0.849 | -1634.760 |  |
| 2a5 | 71 | 17 | 29.631 | 0.029 | 0.862 | 0.787 | -1632.112 |  |
| 2a1 | 71 | 14 | 23.986 | 0.046 | 0.890 | 0.832 | -1631.757 |  |
| 1a3 | 71 | 16 | 28.313 | 0.029 | 0.869 | 0.793 | -1631.430 |  |
| 1a1 | 71 | 11 | 19.450 | 0.053 | 0.908 | 0.858 | -1630.293 |  |
| 1a6 | 71 | 14 | 21.630 | 0.087 | 0.934 | 0.871 | -1622.113 |  |
| 1a4 | 71 | 14 | 26.894 | 0.020 | 0.920 | 0.783 | -1616.849 |  |
| 2a2 | 71 | 12 | 23.931 | 0.021 | 0.928 | 0.799 | -1615.812 |  |
| 2a3 | 71 | 15 | 74.510 | 0.000 | 0.754 | 0.000 | -1583.233 |  |
| 1a2 | 71 | 13 | 71.537 | 0.000 | 0.757 | 0.014 | -1582.206 |  |
| 2b4x | 71 | 21 | 31.767 | 0.062 | 0.885 | 0.806 | -1482.250 |  |
| 1b7x | 71 | 17 | 31.907 | 0.015 | 0.857 | 0.733 | -1482.110 |  |
| 1b3x | 71 | 23 | 37.290 | 0.030 | 0.863 | 0.743 | -1480.727 |  |
| 1b1x | 71 | 20 | 35.401 | 0.018 | 0.866 | 0.723 | -1476.615 |  |
| 2b1x | 71 | 17 | 29.509 | 0.030 | 0.889 | 0.775 | -1476.508 |  |
| 1b5x | 71 | 23 | 43.186 | 0.007 | 0.838 | 0.637 | -1474.831 |  |
| 2b2x | 71 | 15 | 27.769 | 0.023 | 0.893 | 0.770 | -1474.248 |  |
| 2b5x | 71 | 21 | 40.586 | 0.006 | 0.850 | 0.648 | -1473.430 |  |
| 1b8x | 71 | 14 | 27.794 | 0.015 | 0.894 | 0.752 | -1472.223 |  |
| 1b10x | 71 | 18 | 33.456 | 0.015 | 0.909 | 0.722 | -1462.560 |  |
| 1b6x | 71 | 21 | 39.836 | 0.008 | 0.894 | 0.661 | -1462.181 |  |
| 2b4 | 71 | 21 | 33.138 | 0.045 | 0.873 | 0.824 | -1461.276 |  |
| 1b7 | 71 | 17 | 33.602 | 0.009 | 0.852 | 0.760 | -1460.813 |  |
| 1b9x | 71 | 18 | 35.242 | 0.009 | 0.909 | 0.690 | -1460.775 |  |
| 1b3 | 71 | 23 | 38.515 | 0.022 | 0.853 | 0.775 | -1459.899 |  |
| 1b5 | 71 | 23 | 39.044 | 0.020 | 0.854 | 0.768 | -1459.371 |  |
| 2b5 | 71 | 21 | 36.121 | 0.021 | 0.865 | 0.781 | -1458.293 |  |
| 1b11 | 71 | 11 | 24.177 | 0.012 | 0.889 | 0.810 | -1458.238 |  |
| 1b1 | 71 | 20 | 36.069 | 0.015 | 0.865 | 0.767 | -1456.346 |  |
| 2b1 | 71 | 17 | 30.322 | 0.024 | 0.886 | 0.807 | -1456.093 |  |
| 1b8 | 71 | 14 | 26.644 | 0.021 | 0.897 | 0.817 | -1453.771 |  |
| 2b2 | 71 | 15 | 30.212 | 0.011 | 0.885 | 0.780 | -1452.203 |  |
| 1b10 | 71 | 18 | 28.911 | 0.049 | 0.922 | 0.842 | -1447.504 |  |
| 1b2x | 71 | 22 | 68.771 | 0.000 | 0.782 | 0.159 | -1447.246 |  |
| 1b6 | 71 | 21 | 36.460 | 0.019 | 0.904 | 0.776 | -1445.955 |  |
| 2b3x | 71 | 18 | 62.086 | 0.000 | 0.800 | 0.207 | -1445.931 |  |
| 1b4 | 71 | 21 | 37.097 | 0.016 | 0.903 | 0.767 | -1445.318 |  |
| 1b9 | 71 | 18 | 34.091 | 0.012 | 0.911 | 0.767 | -1442.323 |  |
| 1b2 | 71 | 22 | 87.071 | 0.000 | 0.757 | 0.058 | -1409.344 |  |
| 2b3 | 71 | 18 | 80.835 | 0.000 | 0.772 | 0.090 | -1407.579 |  |
| 1b4x | 71 | 30 | 85.883 | 0.000 | 0.824 | 0.510 | -1386.382 |  |
| 1b11x | 71 | 14 | 1154.559 | 0.000 | 0.672 | 0.037 | -1315.064 |  |
| *Tarsus Asymmetry: Fringilla coelebs* | | | | | | | | |
| **Model name** | **n** | **dF** | **Chisq** | **P-value** | **GFI** | **CFI** | **AIC** | **ΔAIC** |
| 1a7 | 41 | 8 | 11.470 | 0.176 | 0.888 | 0.880 | -825.146 |  |
| 2a5 | 41 | 17 | 21.579 | 0.201 | 0.838 | 0.838 | -825.037 |  |
| 1a5 | 41 | 16 | 20.117 | 0.215 | 0.846 | 0.854 | -824.499 |  |
| 2a4 | 41 | 17 | 24.368 | 0.110 | 0.819 | 0.739 | -822.248 |  |
| 1a3 | 41 | 16 | 22.542 | 0.127 | 0.832 | 0.768 | -822.074 |  |
| 2a1 | 41 | 14 | 19.603 | 0.143 | 0.855 | 0.802 | -821.013 |  |
| 1a1 | 41 | 11 | 13.674 | 0.252 | 0.894 | 0.905 | -820.943 |  |
| 1a4 | 41 | 14 | 17.983 | 0.208 | 0.911 | 0.859 | -810.633 |  |
| 1a6 | 41 | 14 | 18.400 | 0.189 | 0.909 | 0.844 | -810.216 |  |
| 2a2 | 41 | 12 | 15.013 | 0.241 | 0.922 | 0.893 | -809.604 |  |
| 2a3 | 41 | 15 | 35.557 | 0.002 | 0.757 | 0.272 | -807.059 |  |
| 1a2 | 41 | 13 | 33.345 | 0.002 | 0.782 | 0.280 | -805.272 |  |
| 1b7 | 41 | 17 | 33.323 | 0.010 | 0.786 | 0.668 | -730.954 |  |
| 1b5 | 41 | 23 | 37.417 | 0.029 | 0.798 | 0.703 | -730.859 |  |
| 1b7x | 41 | 17 | 27.723 | 0.048 | 0.808 | 0.851 | -729.277 |  |
| 2b5 | 41 | 21 | 36.924 | 0.017 | 0.801 | 0.672 | -727.353 |  |
| 1b11 | 41 | 11 | 25.060 | 0.009 | 0.833 | 0.714 | -727.216 |  |
| 1b1 | 41 | 20 | 35.527 | 0.017 | 0.805 | 0.680 | -726.750 |  |
| 1b3 | 41 | 23 | 42.156 | 0.009 | 0.765 | 0.605 | -726.121 |  |
| 1b1x | 41 | 20 | 29.225 | 0.083 | 0.822 | 0.871 | -725.775 |  |
| 2b1x | 41 | 17 | 25.758 | 0.079 | 0.843 | 0.878 | -723.242 |  |
| 1b8 | 41 | 14 | 27.264 | 0.018 | 0.845 | 0.727 | -723.012 |  |
| 1b3x | 41 | 23 | 38.173 | 0.024 | 0.783 | 0.788 | -722.827 |  |
| 2b4 | 41 | 21 | 41.516 | 0.005 | 0.769 | 0.577 | -722.761 |  |
| 1b8x | 41 | 14 | 20.349 | 0.120 | 0.872 | 0.911 | -722.651 |  |
| 2b4x | 41 | 21 | 34.760 | 0.030 | 0.801 | 0.808 | -722.240 |  |
| 2b2 | 41 | 15 | 30.145 | 0.011 | 0.828 | 0.688 | -722.132 |  |
| 2b1 | 41 | 17 | 34.832 | 0.007 | 0.810 | 0.633 | -721.445 |  |
| 2b2x | 41 | 15 | 23.678 | 0.071 | 0.854 | 0.879 | -721.322 |  |
| 1b2x | 41 | 22 | 41.317 | 0.008 | 0.766 | 0.730 | -717.683 |  |
| 1b6 | 41 | 21 | 35.700 | 0.024 | 0.859 | 0.697 | -716.577 |  |
| 1b4 | 41 | 21 | 37.597 | 0.014 | 0.854 | 0.658 | -714.680 |  |
| 2b3x | 41 | 18 | 37.475 | 0.005 | 0.787 | 0.728 | -713.525 |  |
| 1b10 | 41 | 18 | 32.754 | 0.018 | 0.868 | 0.696 | -713.522 |  |
| 1b9 | 41 | 18 | 35.393 | 0.008 | 0.862 | 0.642 | -710.883 |  |
| 1b9x | 41 | 18 | 28.473 | 0.055 | 0.880 | 0.854 | -710.527 |  |
| 1b2 | 41 | 22 | 57.582 | 0.000 | 0.699 | 0.267 | -708.695 |  |
| 2b3 | 41 | 18 | 50.968 | 0.000 | 0.733 | 0.321 | -707.309 |  |
| 1b6x | 41 | 21 | 40.194 | 0.007 | 0.845 | 0.732 | -704.806 |  |
| 1b10x | 41 | 18 | 34.437 | 0.011 | 0.865 | 0.771 | -704.563 |  |
| 1b5x | 41 | 23 | 66.016 | 0.000 | 0.680 | 0.400 | -694.984 |  |
| 2b5x | 41 | 21 | 64.445 | 0.000 | 0.684 | 0.394 | -692.555 |  |
| 1b4x | 41 | 30 | 67.658 | 0.000 | 0.784 | 0.639 | -692.383 |  |
| 1b11x | 41 | 14 | 558.336 | 0.000 | 0.671 | 0.109 | -637.025 |  |
| *Tarsus Asymmetry: Parus major* | | | | | | | | |
| **Model name** | **n** | **dF** | **Chisq** | **P-value** | **GFI** | **CFI** | **AIC** | **ΔAIC** |
| 2a5 | 84 | 17 | 14.571 | 0.626 | 0.936 | 1.000 | -1801.672 | 0.000 |
| 2a4 | 84 | 17 | 14.994 | 0.596 | 0.939 | 1.000 | -1801.249 | 0.423 |
| 1a5 | 84 | 16 | 13.060 | 0.668 | 0.942 | 1.000 | -1801.184 | 0.488 |
| 2a1 | 84 | 14 | 10.180 | 0.749 | 0.955 | 1.000 | -1800.064 | 1.608 |
| 1a3 | 84 | 16 | 16.575 | 0.414 | 0.930 | 0.983 | -1797.668 | 4.004 |
| 1a7 | 84 | 8 | 10.475 | 0.233 | 0.943 | 0.932 | -1795.768 | 5.904 |
| 1a1 | 84 | 11 | 12.433 | 0.332 | 0.946 | 0.958 | -1791.810 | 9.862 |
| 2a2 | 84 | 12 | 8.199 | 0.769 | 0.977 | 1.000 | -1786.045 | 15.627 |
| 1a6 | 84 | 14 | 12.812 | 0.541 | 0.964 | 1.000 | -1785.432 | 16.241 |
| 1a4 | 84 | 14 | 13.837 | 0.462 | 0.962 | 1.000 | -1784.406 | 17.266 |
| 2a3 | 84 | 15 | 45.104 | 0.000 | 0.844 | 0.127 | -1767.139 |  |
| 1a2 | 84 | 13 | 52.525 | 0.000 | 0.813 | 0.000 | -1755.718 |  |
| 1b5 | 84 | 23 | 28.114 | 0.211 | 0.903 | 0.879 | -1612.186 |  |
| 1b7 | 84 | 17 | 25.200 | 0.090 | 0.898 | 0.813 | -1611.100 |  |
| 1b3 | 84 | 23 | 30.944 | 0.124 | 0.895 | 0.812 | -1609.356 |  |
| 2b5 | 84 | 21 | 28.406 | 0.129 | 0.905 | 0.824 | -1607.895 |  |
| 1b1 | 84 | 20 | 27.158 | 0.131 | 0.908 | 0.830 | -1607.143 |  |
| 2b4 | 84 | 21 | 29.199 | 0.109 | 0.905 | 0.806 | -1607.102 |  |
| 2b1 | 84 | 17 | 23.777 | 0.126 | 0.921 | 0.839 | -1604.524 |  |
| 2b2 | 84 | 15 | 21.272 | 0.128 | 0.927 | 0.851 | -1603.029 |  |
| 1b11 | 84 | 11 | 23.675 | 0.014 | 0.904 | 0.711 | -1600.625 |  |
| 1b8 | 84 | 14 | 25.633 | 0.029 | 0.913 | 0.724 | -1596.668 |  |
| 1b6 | 84 | 21 | 27.867 | 0.144 | 0.934 | 0.837 | -1596.434 |  |
| 1b4 | 84 | 21 | 28.206 | 0.134 | 0.933 | 0.829 | -1596.094 |  |
| 1b10 | 84 | 18 | 26.634 | 0.086 | 0.936 | 0.795 | -1591.666 |  |
| 1b9 | 84 | 18 | 27.972 | 0.062 | 0.934 | 0.763 | -1590.329 |  |
| 2b3 | 84 | 18 | 58.465 | 0.000 | 0.834 | 0.040 | -1571.836 |  |
| 1b2 | 84 | 22 | 66.757 | 0.000 | 0.809 | 0.000 | -1571.544 |  |
| 1b7x | 83 | 17 | 25.347 | 0.087 | 0.894 | 0.750 | -1525.661 |  |
| 1b3x | 83 | 23 | 30.421 | 0.138 | 0.894 | 0.767 | -1524.588 |  |
| 1b1x | 83 | 20 | 27.630 | 0.118 | 0.905 | 0.761 | -1521.379 |  |
| 2b4x | 83 | 21 | 30.450 | 0.083 | 0.898 | 0.703 | -1520.559 |  |
| 2b1x | 83 | 17 | 25.854 | 0.077 | 0.912 | 0.722 | -1517.155 |  |
| 2b2x | 83 | 15 | 23.056 | 0.083 | 0.918 | 0.747 | -1515.953 |  |
| 1b8x | 83 | 14 | 22.760 | 0.064 | 0.921 | 0.725 | -1514.248 |  |
| 1b5x | 83 | 23 | 42.266 | 0.008 | 0.861 | 0.395 | -1512.742 |  |
| 1b2x | 83 | 22 | 42.197 | 0.006 | 0.861 | 0.366 | -1510.811 |  |
| 2b5x | 83 | 21 | 41.267 | 0.005 | 0.866 | 0.364 | -1509.742 |  |
| 2b3x | 83 | 18 | 37.067 | 0.005 | 0.876 | 0.402 | -1507.941 |  |
| 1b9x | 83 | 18 | 25.330 | 0.116 | 0.939 | 0.770 | -1507.679 |  |
| 1b6x | 83 | 21 | 35.937 | 0.022 | 0.921 | 0.531 | -1503.071 |  |
| 1b10x | 83 | 18 | 35.023 | 0.009 | 0.923 | 0.466 | -1497.986 |  |
| 1b4x | 83 | 30 | 64.709 | 0.000 | 0.877 | 0.589 | -1466.442 |  |
| 1b11x | 83 | 14 | 1164.236 | 0.000 | 0.696 | 0.026 | -1343.535 |  |
| *Tarsus Asymmetry: Sylvia atricapilla* | | | | | | | | |
| **Model name** | **n** | **dF** | **Chisq** | **P-value** | **GFI** | **CFI** | **AIC** | **ΔAIC** |
| 1a5 | 61 | 16 | 19.251 | 0.256 | 0.899 | 0.890 | -1395.270 |  |
| 1a7 | 61 | 8 | 13.668 | 0.091 | 0.906 | 0.823 | -1392.854 |  |
| 1a1 | 61 | 11 | 15.007 | 0.182 | 0.918 | 0.864 | -1389.514 |  |
| 1a3 | 61 | 16 | 27.583 | 0.035 | 0.861 | 0.607 | -1386.938 |  |
| 2a1 | 61 | 14 | 26.274 | 0.024 | 0.867 | 0.584 | -1384.247 |  |
| 2a4 | 61 | 17 | 33.232 | 0.011 | 0.841 | 0.449 | -1383.290 |  |
| 1a6 | 61 | 14 | 16.418 | 0.289 | 0.943 | 0.918 | -1382.103 | 0.000 |
| 2a5 | 61 | 17 | 35.027 | 0.006 | 0.828 | 0.388 | -1381.494 |  |
| 2a3 | 61 | 15 | 37.977 | 0.001 | 0.835 | 0.220 | -1374.545 |  |
| 1a4 | 61 | 14 | 25.688 | 0.028 | 0.915 | 0.603 | -1372.833 |  |
| 1a2 | 61 | 13 | 36.512 | 0.000 | 0.810 | 0.202 | -1372.009 |  |
| 2a2 | 61 | 12 | 23.130 | 0.027 | 0.922 | 0.622 | -1371.392 |  |
| 1b11 | 61 | 11 | 24.302 | 0.012 | 0.876 | 0.656 | -1225.348 |  |
| 1b8 | 61 | 14 | 25.642 | 0.029 | 0.893 | 0.678 | -1222.009 |  |
| 1b3 | 61 | 23 | 46.428 | 0.003 | 0.824 | 0.352 | -1219.222 |  |
| 1b7 | 61 | 17 | 42.975 | 0.000 | 0.801 | 0.329 | -1218.676 |  |
| 1b5 | 61 | 23 | 47.462 | 0.002 | 0.821 | 0.323 | -1218.188 |  |
| 2b4 | 61 | 21 | 44.161 | 0.002 | 0.837 | 0.359 | -1217.489 |  |
| 2b1 | 61 | 17 | 36.557 | 0.004 | 0.853 | 0.459 | -1217.093 |  |
| 1b10 | 61 | 18 | 27.266 | 0.074 | 0.923 | 0.744 | -1216.385 |  |
| 2b2 | 61 | 15 | 34.125 | 0.003 | 0.857 | 0.471 | -1215.526 |  |
| 2b5 | 61 | 21 | 46.181 | 0.001 | 0.822 | 0.303 | -1215.469 |  |
| 1b1 | 61 | 20 | 44.314 | 0.001 | 0.829 | 0.327 | -1215.336 |  |
| 2b3 | 61 | 18 | 47.825 | 0.000 | 0.827 | 0.175 | -1207.826 |  |
| 1b9 | 61 | 18 | 37.052 | 0.005 | 0.899 | 0.473 | -1206.598 |  |
| 1b4 | 61 | 21 | 44.533 | 0.002 | 0.880 | 0.349 | -1205.117 |  |
| 1b6 | 61 | 21 | 44.629 | 0.002 | 0.880 | 0.346 | -1205.022 |  |
| 1b2 | 61 | 22 | 59.435 | 0.000 | 0.785 | 0.000 | -1204.216 |  |
| 1b3x | 60 | 23 | 42.333 | 0.008 | 0.835 | 0.072 | -1124.567 |  |
| 1b7x | 60 | 17 | 39.196 | 0.002 | 0.815 | 0.051 | -1123.704 |  |
| 1b8x | 60 | 14 | 25.871 | 0.027 | 0.890 | 0.430 | -1123.028 |  |
| 1b5x | 60 | 23 | 44.184 | 0.005 | 0.835 | 0.000 | -1122.716 |  |
| 2b5x | 60 | 21 | 40.281 | 0.007 | 0.847 | 0.075 | -1122.619 |  |
| 1b2x | 60 | 22 | 44.063 | 0.003 | 0.829 | 0.000 | -1120.837 |  |
| 2b4x | 60 | 21 | 42.181 | 0.004 | 0.840 | 0.000 | -1120.719 |  |
| 1b1x | 60 | 20 | 40.975 | 0.004 | 0.839 | 0.000 | -1119.925 |  |
| 2b3x | 60 | 18 | 37.308 | 0.005 | 0.852 | 0.074 | -1119.592 |  |
| 2b1x | 60 | 17 | 35.383 | 0.006 | 0.855 | 0.118 | -1119.517 |  |
| 2b2x | 60 | 15 | 32.077 | 0.006 | 0.862 | 0.181 | -1118.823 |  |
| 1b10x | 60 | 18 | 27.571 | 0.069 | 0.920 | 0.541 | -1117.329 |  |
| 1b4x | 60 | 30 | 70.267 | 0.000 | 0.832 | 0.252 | -1117.000 |  |
| 1b6x | 60 | 21 | 42.069 | 0.004 | 0.885 | 0.000 | -1108.831 |  |
| 1b9x | 60 | 18 | 38.183 | 0.004 | 0.894 | 0.032 | -1106.717 |  |
| 1b11x | 60 | 14 | 855.015 | 0.000 | 0.680 | 0.022 | -981.500 |  |
| *Tarsus Asymmetry: Turdus merula* | | | | | | | | |
| **Model name** | **n** | **dF** | **Chisq** | **P-value** | **GFI** | **CFI** | **AIC** | **ΔAIC** |
| 1a3 | 58 | 16 | 8.286 | 0.940 | 0.945 | 1.000 | -1346.260 | 0.000 |
| 1a5 | 58 | 16 | 12.667 | 0.697 | 0.924 | 1.000 | -1341.879 | 4.381 |
| 1a7 | 58 | 8 | 4.705 | 0.789 | 0.962 | 1.000 | -1341.841 | 4.419 |
| 2a4 | 58 | 17 | 16.017 | 0.523 | 0.905 | 1.000 | -1340.529 | 5.731 |
| 1a2 | 58 | 13 | 10.131 | 0.683 | 0.935 | 1.000 | -1338.414 | 7.845 |
| 2a5 | 58 | 17 | 18.394 | 0.364 | 0.893 | 0.000 | -1338.152 |  |
| 1a1 | 58 | 11 | 6.545 | 0.835 | 0.958 | 1.000 | -1338.001 | 8.259 |
| 2a1 | 58 | 14 | 13.661 | 0.475 | 0.919 | 1.000 | -1336.885 | 9.375 |
| 2a3 | 58 | 15 | 15.945 | 0.386 | 0.908 | 0.000 | -1336.601 |  |
| 1a4 | 58 | 14 | 7.065 | 0.932 | 0.970 | 1.000 | -1331.481 | 14.778 |
| 1a6 | 58 | 14 | 8.103 | 0.884 | 0.968 | 1.000 | -1330.443 | 15.817 |
| 2a2 | 58 | 12 | 4.972 | 0.959 | 0.979 | 1.000 | -1329.574 | 16.686 |
| 1b3 | 58 | 23 | 24.224 | 0.392 | 0.891 | 0.870 | -1204.584 |  |
| 1b7 | 58 | 17 | 20.370 | 0.256 | 0.886 | 0.681 | -1204.437 |  |
| 1b2 | 58 | 22 | 24.899 | 0.302 | 0.887 | 0.691 | -1201.908 |  |
| 1b1 | 58 | 20 | 22.210 | 0.329 | 0.895 | 0.764 | -1200.597 |  |
| 1b5 | 58 | 23 | 29.814 | 0.155 | 0.859 | 0.273 | -1198.993 |  |
| 2b4 | 58 | 21 | 29.578 | 0.101 | 0.862 | 0.085 | -1195.229 |  |
| 1b11 | 58 | 11 | 17.847 | 0.085 | 0.900 | 0.352 | -1194.961 |  |
| 2b2 | 58 | 15 | 18.133 | 0.256 | 0.913 | 0.666 | -1194.674 |  |
| 2b3 | 58 | 18 | 27.387 | 0.072 | 0.866 | 0.000 | -1191.420 |  |
| 1b8 | 58 | 14 | 19.687 | 0.140 | 0.907 | 0.394 | -1191.121 |  |
| 2b1 | 58 | 17 | 25.787 | 0.078 | 0.872 | 0.063 | -1191.021 |  |
| 2b5 | 58 | 21 | 34.164 | 0.035 | 0.836 | 0.000 | -1190.644 |  |
| 1b4 | 58 | 21 | 23.002 | 0.344 | 0.926 | 0.787 | -1189.805 |  |
| 1b6 | 58 | 21 | 25.250 | 0.236 | 0.920 | 0.547 | -1187.557 |  |
| 1b9 | 58 | 18 | 21.121 | 0.273 | 0.932 | 0.667 | -1185.687 |  |
| 1b10 | 58 | 18 | 24.254 | 0.147 | 0.925 | 0.333 | -1182.553 |  |
| 1b4x | 57 | 30 | 42.970 | 0.059 | 0.881 | 0.159 | -1155.348 |  |
| 1b3x | 57 | 23 | 22.200 | 0.508 | 0.898 | 1.000 | -1100.791 |  |
| 1b5x | 57 | 23 | 22.968 | 0.463 | 0.889 | 1.000 | -1100.023 |  |
| 1b7x | 57 | 17 | 19.083 | 0.324 | 0.892 | 0.010 | -1099.908 |  |
| 1b2x | 57 | 22 | 22.165 | 0.450 | 0.899 | 0.792 | -1098.826 |  |
| 1b1x | 57 | 20 | 20.907 | 0.403 | 0.900 | 0.000 | -1096.083 |  |
| 2b5x | 57 | 21 | 27.504 | 0.155 | 0.862 | 0.000 | -1091.487 |  |
| 2b4x | 57 | 21 | 28.223 | 0.134 | 0.865 | 0.000 | -1090.768 |  |
| 2b2x | 57 | 15 | 17.179 | 0.308 | 0.917 | 0.000 | -1089.812 |  |
| 2b3x | 57 | 18 | 25.738 | 0.106 | 0.871 | 0.000 | -1087.253 |  |
| 2b1x | 57 | 17 | 25.413 | 0.086 | 0.872 | 0.000 | -1085.577 |  |
| 1b8x | 57 | 14 | 19.416 | 0.150 | 0.908 | 0.000 | -1085.574 |  |
| 1b6x | 57 | 21 | 21.504 | 0.429 | 0.929 | 0.365 | -1085.487 |  |
| 1b9x | 57 | 18 | 20.627 | 0.299 | 0.933 | 0.000 | -1080.364 |  |
| 1b10x | 57 | 18 | 20.755 | 0.292 | 0.932 | 0.000 | -1080.235 |  |
| 1b11x | 57 | 14 | 764.092 | 0.000 | 0.701 | 0.004 | -963.525 |  |
| *Tarsus Asymmetry: Turdus philomelos* | | | | | | | | |
| **Model name** | **n** | **dF** | **Chisq** | **P-value** | **GFI** | **CFI** | **AIC** | **ΔAIC** |
| 1a3 | 39 | 16 | 22.133 | 0.139 | 0.840 | 0.667 | -950.421 |  |
| 2a4 | 39 | 17 | 26.680 | 0.063 | 0.815 | 0.474 | -947.874 |  |
| 1a5 | 39 | 16 | 27.473 | 0.037 | 0.798 | 0.376 | -945.081 |  |
| 2a5 | 39 | 17 | 30.436 | 0.023 | 0.779 | 0.270 | -944.117 |  |
| 1a7 | 39 | 8 | 20.907 | 0.007 | 0.815 | 0.386 | -943.647 |  |
| 2a1 | 39 | 14 | 26.059 | 0.025 | 0.816 | 0.345 | -942.495 |  |
| 1a1 | 39 | 11 | 21.233 | 0.031 | 0.844 | 0.444 | -941.320 |  |
| 2a3 | 39 | 15 | 33.175 | 0.004 | 0.782 | 0.012 | -937.379 |  |
| 1a4 | 39 | 14 | 21.549 | 0.088 | 0.895 | 0.590 | -935.005 |  |
| 1a6 | 39 | 14 | 23.156 | 0.058 | 0.888 | 0.502 | -933.398 |  |
| 1a2 | 39 | 13 | 34.299 | 0.001 | 0.769 | 0.000 | -932.255 |  |
| 2a2 | 39 | 12 | 24.723 | 0.016 | 0.882 | 0.308 | -927.830 |  |
| 1b3 | 39 | 23 | 37.380 | 0.030 | 0.804 | 0.450 | -863.432 |  |
| 1b7 | 39 | 17 | 36.335 | 0.004 | 0.773 | 0.328 | -860.477 |  |
| 1b5 | 39 | 23 | 41.520 | 0.010 | 0.772 | 0.292 | -859.293 |  |
| 1b1 | 39 | 20 | 36.662 | 0.013 | 0.803 | 0.363 | -858.150 |  |
| 2b4 | 39 | 21 | 38.694 | 0.011 | 0.799 | 0.323 | -858.119 |  |
| 2b5 | 39 | 21 | 42.867 | 0.003 | 0.768 | 0.164 | -853.946 |  |
| 1b11 | 39 | 11 | 32.803 | 0.001 | 0.793 | 0.242 | -852.010 |  |
| 2b1 | 39 | 17 | 37.932 | 0.003 | 0.798 | 0.199 | -850.880 |  |
| 1b2 | 39 | 22 | 48.298 | 0.001 | 0.741 | 0.000 | -850.514 |  |
| 1b3x | 39 | 23 | 37.816 | 0.027 | 0.800 | 0.580 | -849.861 |  |
| 1b8 | 39 | 14 | 33.129 | 0.003 | 0.820 | 0.268 | -849.683 |  |
| 1b7x | 39 | 17 | 34.753 | 0.007 | 0.776 | 0.532 | -848.924 |  |
| 2b2 | 39 | 15 | 36.614 | 0.001 | 0.803 | 0.173 | -848.198 |  |
| 1b4 | 39 | 21 | 36.796 | 0.018 | 0.859 | 0.396 | -848.016 |  |
| 1b6 | 39 | 21 | 37.202 | 0.016 | 0.857 | 0.380 | -847.610 |  |
| 1b1x | 39 | 20 | 35.683 | 0.017 | 0.801 | 0.556 | -845.994 |  |
| 2b3 | 39 | 18 | 44.914 | 0.000 | 0.771 | 0.000 | -845.898 |  |
| 1b9 | 39 | 18 | 33.616 | 0.014 | 0.870 | 0.403 | -845.196 |  |
| 2b4x | 39 | 21 | 38.999 | 0.010 | 0.797 | 0.490 | -844.678 |  |
| 1b10 | 39 | 18 | 35.581 | 0.008 | 0.863 | 0.328 | -843.231 |  |
| 1b2x | 39 | 22 | 44.057 | 0.003 | 0.752 | 0.375 | -841.621 |  |
| 2b1x | 39 | 17 | 36.823 | 0.004 | 0.798 | 0.439 | -838.855 |  |
| 2b3x | 39 | 18 | 39.263 | 0.003 | 0.787 | 0.398 | -838.415 |  |
| 2b2x | 39 | 15 | 34.972 | 0.002 | 0.805 | 0.434 | -836.705 |  |
| 1b8x | 39 | 14 | 33.411 | 0.003 | 0.818 | 0.450 | -836.266 |  |
| 1b5x | 39 | 23 | 55.225 | 0.000 | 0.718 | 0.087 | -832.452 |  |
| 1b9x | 39 | 18 | 33.898 | 0.013 | 0.869 | 0.550 | -831.779 |  |
| 1b6x | 39 | 21 | 40.915 | 0.006 | 0.842 | 0.436 | -830.763 |  |
| 2b5x | 39 | 21 | 56.573 | 0.000 | 0.716 | 0.000 | -827.104 |  |
| 1b10x | 39 | 18 | 40.271 | 0.002 | 0.845 | 0.369 | -825.407 |  |
| 1b4x | 39 | 30 | 68.246 | 0.000 | 0.789 | 0.397 | -820.886 |  |
| 1b11x | 39 | 14 | 1450.933 | 0.000 | 0.644 | 0.017 | -765.835 |  |

**^1^ Model name:** The first number refers to whether the model is amended with a measure for vector abundance (1: original model, without measure for vector abundance, 2: model including measure for vector abundance). The first letter describes the underlying hypothesis (a: 'top-down'-hypothesis, b: 'bottom-up'-hypothesis, see Background). The number following the letter indicates the model number (not further described). The addition of an 'x' indicated whether that model was modelled using the species-specific parasite-vector relationship (i.e. *Plasmodium/Haemoproteus* infection in relation the abundance of vectors of the families Culicidae/Ceratopogonidae). E.g.: 1b11x indicates the original model (1), model number (11), 'bottom-up'-hypothesis (addition of 'x').

**Table S3.** Test statistics for all models for primary 3 asymmetry (SEM). Statistics that meet the criteria are indicated in gray shading (P > 0.05; GFI > 0.9; CFI > 0.9). The best-fitted model for each species is marked in grey. ΔAIC only given when all criteria were met. For the explanation of the model names, see footnote.

| *Primary 3 Asymmetry: all bird species* | | | | | | | | |
| --- | --- | --- | --- | --- | --- | --- | --- | --- |
| **Model name^1^** | **n** | **dF** | **Chisq** | **P-value** | **GFI** | **CFI** | **AIC** | **ΔAIC** |
| 1a7 | 360 | 8 | 3.045 | 0.931 | 0.996 | 1.000 | -8197.548 | 0.000 |
| 2a1 | 360 | 14 | 19.125 | 0.160 | 0.979 | 0.971 | -8185.468 | 12.079 |
| 1a1 | 360 | 11 | 14.471 | 0.208 | 0.984 | 0.980 | -8184.122 | 13.426 |
| 1a5 | 360 | 16 | 26.440 | 0.048 | 0.972 | 0.941 | -8182.153 |  |
| 2a5 | 360 | 17 | 28.533 | 0.039 | 0.970 | 0.934 | -8182.059 |  |
| 1a6 | 360 | 14 | 17.411 | 0.235 | 0.988 | 0.981 | -8175.182 | 22.366 |
| 2a2 | 360 | 12 | 14.624 | 0.263 | 0.990 | 0.985 | -8173.969 | 23.579 |
| 2a4 | 360 | 17 | 38.192 | 0.002 | 0.961 | 0.880 | -8172.400 |  |
| 1a4 | 360 | 14 | 23.088 | 0.059 | 0.984 | 0.948 | -8169.504 | 28.043 |
| 1a3 | 360 | 16 | 41.751 | 0.000 | 0.957 | 0.854 | -8166.841 |  |
| 2a3 | 360 | 15 | 165.692 | 0.000 | 0.867 | 0.143 | -8040.901 |  |
| 1a2 | 360 | 13 | 162.590 | 0.000 | 0.868 | 0.150 | -8040.002 |  |
| 1b11 | 360 | 11 | 14.200 | 0.222 | 0.985 | 0.983 | -7338.065 | 859.482 |
| 1b7 | 360 | 17 | 26.349 | 0.068 | 0.972 | 0.950 | -7337.916 | 859.631 |
| 2b2 | 360 | 15 | 25.335 | 0.046 | 0.977 | 0.947 | -7326.930 |  |
| 2b1 | 360 | 17 | 29.940 | 0.027 | 0.973 | 0.933 | -7326.325 |  |
| 2b5 | 360 | 21 | 39.572 | 0.008 | 0.965 | 0.904 | -7324.693 |  |
| 1b8 | 360 | 14 | 25.626 | 0.029 | 0.977 | 0.940 | -7324.639 |  |
| 1b1 | 360 | 20 | 37.774 | 0.009 | 0.967 | 0.908 | -7324.490 |  |
| 1b5 | 360 | 23 | 47.077 | 0.002 | 0.959 | 0.876 | -7321.187 |  |
| 1b10 | 360 | 18 | 29.072 | 0.048 | 0.983 | 0.943 | -7317.193 |  |
| 1b6 | 360 | 21 | 38.048 | 0.013 | 0.977 | 0.912 | -7314.217 |  |
| 2b4 | 360 | 21 | 50.283 | 0.000 | 0.958 | 0.849 | -7313.982 |  |
| 1b4 | 360 | 21 | 39.165 | 0.009 | 0.977 | 0.906 | -7313.100 |  |
| 1b9 | 360 | 18 | 35.196 | 0.009 | 0.979 | 0.911 | -7311.069 |  |
| 1b3 | 360 | 23 | 57.828 | 0.000 | 0.951 | 0.820 | -7310.437 |  |
| 2b3 | 360 | 18 | 176.333 | 0.000 | 0.879 | 0.183 | -7181.932 |  |
| 1b2 | 360 | 22 | 184.623 | 0.000 | 0.873 | 0.161 | -7181.642 |  |
| 1b7x | 357 | 17 | 30.439 | 0.023 | 0.967 | 0.890 | -7079.308 |  |
| 2b2x | 357 | 15 | 28.411 | 0.019 | 0.975 | 0.897 | -7069.336 |  |
| 1b8x | 357 | 14 | 27.631 | 0.016 | 0.975 | 0.895 | -7068.116 |  |
| 2b1x | 357 | 17 | 34.584 | 0.007 | 0.969 | 0.865 | -7067.164 |  |
| 1b1x | 357 | 20 | 42.444 | 0.002 | 0.963 | 0.827 | -7065.304 |  |
| 2b4x | 357 | 21 | 49.701 | 0.000 | 0.957 | 0.779 | -7060.047 |  |
| 2b5x | 357 | 21 | 49.710 | 0.000 | 0.957 | 0.779 | -7060.037 |  |
| 1b3x | 357 | 23 | 57.275 | 0.000 | 0.951 | 0.736 | -7056.473 |  |
| 1b5x | 357 | 23 | 57.560 | 0.000 | 0.951 | 0.734 | -7056.188 |  |
| 1b9x | 357 | 18 | 37.719 | 0.004 | 0.977 | 0.848 | -7054.028 |  |
| 1b10x | 357 | 18 | 40.061 | 0.002 | 0.976 | 0.830 | -7051.687 |  |
| 1b6x | 357 | 21 | 49.488 | 0.000 | 0.971 | 0.781 | -7048.259 |  |
| 2b3x | 357 | 18 | 107.686 | 0.000 | 0.915 | 0.310 | -6996.062 |  |
| 1b2x | 357 | 22 | 115.783 | 0.000 | 0.909 | 0.278 | -6995.964 |  |
| 1b4x | 357 | 30 | 270.882 | 0.000 | 0.893 | 0.431 | -6758.734 |  |
| 1b11x | 357 | 14 | 4962.770 | 0.000 | 0.712 | 0.024 | -6249.167 |  |
| *Primary 3 Asymmetry: Cyanistes caeruleus* | | | | | | | | |
| **Model name^1^** | **n** | **dF** | **Chisq** | **P-value** | **GFI** | **CFI** | **AIC** | **ΔAIC** |
| 1a5 | 31 | 16 | 20.636 | 0.193 | 0.811 | 0.816 | -696.268 |  |
| 2a5 | 31 | 17 | 24.886 | 0.097 | 0.792 | 0.688 | -694.018 |  |
| 2a4 | 31 | 17 | 25.481 | 0.084 | 0.784 | 0.664 | -693.423 |  |
| 1a7 | 31 | 8 | 15.550 | 0.049 | 0.830 | 0.708 | -693.354 |  |
| 1a3 | 31 | 16 | 24.654 | 0.076 | 0.778 | 0.657 | -692.250 |  |
| 2a1 | 31 | 14 | 23.096 | 0.059 | 0.806 | 0.640 | -689.808 |  |
| 1a1 | 31 | 11 | 18.898 | 0.063 | 0.833 | 0.687 | -688.006 |  |
| 1a6 | 31 | 14 | 20.584 | 0.113 | 0.873 | 0.739 | -680.320 |  |
| 2a2 | 31 | 12 | 19.053 | 0.087 | 0.886 | 0.721 | -677.851 |  |
| 1a4 | 31 | 14 | 23.120 | 0.058 | 0.860 | 0.639 | -677.784 |  |
| 2a3 | 31 | 15 | 38.352 | 0.001 | 0.716 | 0.075 | -676.552 |  |
| 1a2 | 31 | 13 | 38.346 | 0.000 | 0.681 | 0.000 | -672.558 |  |
| 1b7 | 31 | 17 | 29.295 | 0.032 | 0.766 | 0.653 | -627.120 |  |
| 1b3 | 31 | 23 | 34.422 | 0.059 | 0.760 | 0.673 | -625.993 |  |
| 1b5 | 31 | 23 | 35.341 | 0.048 | 0.753 | 0.646 | -625.074 |  |
| 1b7x | 31 | 17 | 26.856 | 0.060 | 0.780 | 0.712 | -622.013 |  |
| 2b4 | 31 | 21 | 34.608 | 0.031 | 0.780 | 0.610 | -621.807 |  |
| 1b1 | 31 | 20 | 32.644 | 0.037 | 0.771 | 0.638 | -621.771 |  |
| 1b11 | 31 | 11 | 23.660 | 0.014 | 0.803 | 0.643 | -620.755 |  |
| 2b5 | 31 | 21 | 35.802 | 0.023 | 0.771 | 0.576 | -620.613 |  |
| 1b3x | 31 | 23 | 32.675 | 0.087 | 0.768 | 0.713 | -620.194 |  |
| 2b2 | 31 | 15 | 27.409 | 0.026 | 0.806 | 0.645 | -617.006 |  |
| 2b1 | 31 | 17 | 31.808 | 0.016 | 0.796 | 0.576 | -616.607 |  |
| 1b1x | 31 | 20 | 30.312 | 0.065 | 0.786 | 0.694 | -616.557 |  |
| 2b4x | 31 | 21 | 32.672 | 0.050 | 0.782 | 0.654 | -616.197 |  |
| 1b8 | 31 | 14 | 27.008 | 0.019 | 0.812 | 0.627 | -615.407 |  |
| 2b1x | 31 | 17 | 29.287 | 0.032 | 0.808 | 0.635 | -611.582 |  |
| 1b4 | 31 | 21 | 32.888 | 0.047 | 0.835 | 0.659 | -611.527 |  |
| 2b2x | 31 | 15 | 25.953 | 0.039 | 0.813 | 0.675 | -610.915 |  |
| 1b5x | 31 | 23 | 42.185 | 0.009 | 0.721 | 0.431 | -610.683 |  |
| 1b8x | 31 | 14 | 25.636 | 0.029 | 0.822 | 0.655 | -609.233 |  |
| 1b6 | 31 | 21 | 35.289 | 0.026 | 0.820 | 0.591 | -609.126 |  |
| 2b5x | 31 | 21 | 41.161 | 0.005 | 0.746 | 0.402 | -607.707 |  |
| 1b9 | 31 | 18 | 31.290 | 0.027 | 0.842 | 0.619 | -607.125 |  |
| 1b10 | 31 | 18 | 32.229 | 0.021 | 0.834 | 0.592 | -606.186 |  |
| 1b2x | 31 | 22 | 46.883 | 0.002 | 0.678 | 0.262 | -603.985 |  |
| 1b2 | 31 | 22 | 56.829 | 0.000 | 0.647 | 0.002 | -601.586 |  |
| 1b9x | 31 | 18 | 29.917 | 0.038 | 0.849 | 0.646 | -600.951 |  |
| 2b3x | 31 | 18 | 42.103 | 0.001 | 0.732 | 0.285 | -600.765 |  |
| 2b3 | 31 | 18 | 51.197 | 0.000 | 0.709 | 0.049 | -599.218 |  |
| 1b6x | 31 | 21 | 40.917 | 0.006 | 0.805 | 0.409 | -595.951 |  |
| 1b10x | 31 | 18 | 39.120 | 0.003 | 0.816 | 0.373 | -591.748 |  |
| 1b4x | 31 | 30 | 86.473 | 0.000 | 0.734 | 0.331 | -577.754 |  |
| 1b11x | 31 | 14 | 1131.927 | 0.000 | 0.703 | 0.025 | -551.808 |  |
| *Primary 3 Asymmetry: Erithacus rubecula* | | | | | | | | |
| **Model name^1^** | **n** | **dF** | **Chisq** | **P-value** | **GFI** | **CFI** | **AIC** | **ΔAIC** |
| 1a7 | 68 | 8 | 11.920 | 0.155 | 0.925 | 0.928 | -1708.282 | 0.000 |
| 1a5 | 68 | 16 | 21.547 | 0.158 | 0.893 | 0.898 | -1706.656 |  |
| 1a1 | 68 | 11 | 14.695 | 0.197 | 0.925 | 0.932 | -1703.507 | 4.775 |
| 2a4 | 68 | 17 | 27.175 | 0.056 | 0.867 | 0.813 | -1703.027 |  |
| 2a5 | 68 | 17 | 28.370 | 0.041 | 0.863 | 0.792 | -1701.832 |  |
| 1a3 | 68 | 16 | 26.565 | 0.047 | 0.871 | 0.806 | -1701.637 |  |
| 2a1 | 68 | 14 | 25.692 | 0.028 | 0.878 | 0.786 | -1698.510 |  |
| 1a6 | 68 | 14 | 18.976 | 0.166 | 0.938 | 0.909 | -1693.226 | 15.056 |
| 1a4 | 68 | 14 | 25.181 | 0.033 | 0.921 | 0.795 | -1687.021 |  |
| 2a2 | 68 | 12 | 25.116 | 0.014 | 0.922 | 0.759 | -1683.086 |  |
| 1a2 | 68 | 13 | 60.952 | 0.000 | 0.775 | 0.121 | -1661.250 |  |
| 2a3 | 68 | 15 | 73.234 | 0.000 | 0.741 | 0.000 | -1652.968 |  |
| 2b4x | 68 | 21 | 33.347 | 0.043 | 0.872 | 0.782 | -1561.271 |  |
| 1b7x | 68 | 17 | 34.480 | 0.007 | 0.842 | 0.692 | -1560.138 |  |
| 1b3x | 68 | 23 | 40.895 | 0.012 | 0.850 | 0.683 | -1557.723 |  |
| 2b1x | 68 | 17 | 30.980 | 0.020 | 0.877 | 0.753 | -1555.638 |  |
| 1b1x | 68 | 20 | 38.700 | 0.007 | 0.853 | 0.669 | -1553.918 |  |
| 2b2x | 68 | 15 | 29.554 | 0.014 | 0.882 | 0.743 | -1553.064 |  |
| 1b8x | 68 | 14 | 27.826 | 0.015 | 0.889 | 0.755 | -1552.792 |  |
| 1b5x | 68 | 23 | 47.611 | 0.002 | 0.831 | 0.565 | -1551.007 |  |
| 2b5x | 68 | 21 | 44.527 | 0.002 | 0.840 | 0.584 | -1550.091 |  |
| 1b10x | 68 | 18 | 36.912 | 0.005 | 0.904 | 0.666 | -1539.706 |  |
| 1b7 | 68 | 17 | 28.303 | 0.042 | 0.867 | 0.813 | -1539.095 |  |
| 1b11 | 68 | 11 | 16.339 | 0.129 | 0.919 | 0.911 | -1539.059 | 169.223 |
| 1b6x | 68 | 21 | 43.978 | 0.002 | 0.887 | 0.594 | -1538.640 |  |
| 1b9x | 68 | 18 | 38.501 | 0.003 | 0.896 | 0.637 | -1538.117 |  |
| 1b3 | 68 | 23 | 33.909 | 0.066 | 0.864 | 0.818 | -1537.489 |  |
| 1b5 | 68 | 23 | 34.424 | 0.059 | 0.865 | 0.810 | -1536.974 |  |
| 2b4 | 68 | 21 | 32.024 | 0.058 | 0.870 | 0.816 | -1535.374 |  |
| 2b5 | 68 | 21 | 32.527 | 0.052 | 0.871 | 0.808 | -1534.871 |  |
| 1b1 | 68 | 20 | 31.078 | 0.054 | 0.877 | 0.816 | -1534.320 |  |
| 1b8 | 68 | 14 | 19.114 | 0.161 | 0.921 | 0.915 | -1534.284 | 173.998 |
| 2b1 | 68 | 17 | 29.021 | 0.034 | 0.884 | 0.800 | -1530.377 |  |
| 1b2x | 68 | 22 | 69.276 | 0.000 | 0.771 | 0.164 | -1527.342 |  |
| 2b2 | 68 | 15 | 28.520 | 0.019 | 0.886 | 0.775 | -1526.878 |  |
| 2b3x | 68 | 18 | 62.176 | 0.000 | 0.786 | 0.219 | -1526.442 |  |
| 1b10 | 68 | 18 | 23.827 | 0.161 | 0.932 | 0.903 | -1525.571 | 182.711 |
| 1b6 | 68 | 21 | 31.853 | 0.061 | 0.912 | 0.819 | -1523.545 |  |
| 1b4 | 68 | 21 | 32.525 | 0.052 | 0.911 | 0.808 | -1522.873 |  |
| 1b9 | 68 | 18 | 29.788 | 0.040 | 0.918 | 0.804 | -1519.610 |  |
| 1b2 | 68 | 22 | 78.349 | 0.000 | 0.762 | 0.062 | -1491.049 |  |
| 2b3 | 68 | 18 | 75.737 | 0.000 | 0.768 | 0.039 | -1485.661 |  |
| 1b4x | 68 | 30 | 86.224 | 0.000 | 0.822 | 0.494 | -1465.396 |  |
| 1b11x | 68 | 14 | 1112.923 | 0.000 | 0.664 | 0.039 | -1400.051 |  |
| *Primary 3 Asymmetry: Fringilla coelebs* | | | | | | | | |
| **Model name^1^** | **n** | **dF** | **Chisq** | **P-value** | **GFI** | **CFI** | **AIC** | **ΔAIC** |
| 2a5 | 41 | 17 | 15.638 | 0.550 | 0.872 | 1.000 | -927.332 |  |
| 2a4 | 41 | 17 | 16.892 | 0.462 | 0.862 | 1.000 | -926.077 |  |
| 1a7 | 41 | 8 | 7.872 | 0.446 | 0.917 | 1.000 | -925.098 | 0.000 |
| 2a1 | 41 | 14 | 13.677 | 0.474 | 0.889 | 1.000 | -923.292 |  |
| 1a5 | 41 | 16 | 17.745 | 0.339 | 0.866 | 0.915 | -923.225 |  |
| 1a3 | 41 | 16 | 21.330 | 0.166 | 0.837 | 0.741 | -919.640 |  |
| 1a1 | 41 | 11 | 14.407 | 0.211 | 0.887 | 0.834 | -916.563 |  |
| 2a3 | 41 | 15 | 27.140 | 0.028 | 0.805 | 0.409 | -911.830 |  |
| 2a2 | 41 | 12 | 10.678 | 0.557 | 0.942 | 1.000 | -910.292 | 14.806 |
| 1a6 | 41 | 14 | 16.470 | 0.286 | 0.920 | 0.880 | -908.500 |  |
| 1a4 | 41 | 14 | 18.115 | 0.202 | 0.908 | 0.800 | -906.855 |  |
| 1a2 | 41 | 13 | 32.230 | 0.002 | 0.788 | 0.065 | -902.740 |  |
| 1b7 | 41 | 17 | 28.194 | 0.043 | 0.809 | 0.701 | -832.227 |  |
| 1b7x | 41 | 17 | 21.989 | 0.185 | 0.840 | 0.926 | -831.228 |  |
| 2b5 | 41 | 21 | 30.815 | 0.077 | 0.821 | 0.759 | -829.606 |  |
| 1b5 | 41 | 23 | 36.299 | 0.038 | 0.803 | 0.674 | -828.123 |  |
| 1b11 | 41 | 11 | 21.184 | 0.032 | 0.850 | 0.728 | -827.238 |  |
| 2b4 | 41 | 21 | 33.927 | 0.037 | 0.797 | 0.683 | -826.495 |  |
| 1b3 | 41 | 23 | 39.810 | 0.016 | 0.776 | 0.587 | -824.612 |  |
| 2b1 | 41 | 17 | 28.729 | 0.037 | 0.831 | 0.712 | -823.693 |  |
| 1b1 | 41 | 20 | 34.729 | 0.022 | 0.808 | 0.638 | -823.693 |  |
| 2b4x | 41 | 21 | 29.914 | 0.094 | 0.823 | 0.873 | -823.303 |  |
| 2b2 | 41 | 15 | 26.020 | 0.038 | 0.850 | 0.730 | -822.402 |  |
| 2b1x | 41 | 17 | 23.689 | 0.128 | 0.850 | 0.905 | -821.528 |  |
| 1b1x | 41 | 20 | 29.761 | 0.074 | 0.814 | 0.861 | -821.456 |  |
| 1b3x | 41 | 23 | 35.869 | 0.043 | 0.791 | 0.817 | -821.348 |  |
| 2b2x | 41 | 15 | 21.708 | 0.116 | 0.864 | 0.905 | -819.510 |  |
| 1b8x | 41 | 14 | 19.946 | 0.132 | 0.871 | 0.916 | -819.271 |  |
| 1b8 | 41 | 14 | 27.719 | 0.016 | 0.840 | 0.663 | -818.703 |  |
| 1b2x | 41 | 22 | 40.827 | 0.009 | 0.760 | 0.733 | -814.390 |  |
| 2b3x | 41 | 18 | 33.470 | 0.015 | 0.803 | 0.780 | -813.748 |  |
| 1b6 | 41 | 21 | 35.024 | 0.028 | 0.860 | 0.656 | -813.398 |  |
| 2b3 | 41 | 18 | 42.278 | 0.001 | 0.765 | 0.404 | -812.144 |  |
| 1b4 | 41 | 21 | 36.595 | 0.019 | 0.858 | 0.617 | -811.827 |  |
| 1b10 | 41 | 18 | 30.763 | 0.031 | 0.876 | 0.687 | -811.659 |  |
| 1b2 | 41 | 22 | 53.088 | 0.000 | 0.718 | 0.237 | -809.334 |  |
| 1b9x | 41 | 18 | 27.584 | 0.069 | 0.880 | 0.864 | -807.633 |  |
| 1b9 | 41 | 18 | 35.362 | 0.009 | 0.860 | 0.574 | -807.060 |  |
| 1b6x | 41 | 21 | 41.254 | 0.005 | 0.841 | 0.712 | -799.963 |  |
| 1b10x | 41 | 18 | 35.547 | 0.008 | 0.860 | 0.751 | -799.670 |  |
| 1b4x | 41 | 30 | 69.532 | 0.000 | 0.788 | 0.615 | -794.213 |  |
| 2b5x | 41 | 21 | 64.236 | 0.000 | 0.690 | 0.386 | -788.981 |  |
| 1b5x | 41 | 23 | 69.042 | 0.000 | 0.675 | 0.346 | -788.175 |  |
| 1b11x | 41 | 14 | 551.482 | 0.000 | 0.696 | 0.112 | -739.395 |  |
| *Primary 3 Asymmetry: Parus major* | | | | | | | | |
| **Model name^1^** | **n** | **dF** | **Chisq** | **P-value** | **GFI** | **CFI** | **AIC** | **ΔAIC** |
| 2a5 | 81 | 17 | 10.869 | 0.863 | 0.950 | 1.000 | -1952.782 | 0.000 |
| 2a4 | 81 | 17 | 12.021 | 0.799 | 0.945 | 1.000 | -1951.630 | 1.152 |
| 1a5 | 81 | 16 | 12.282 | 0.724 | 0.945 | 1.000 | -1949.370 | 3.412 |
| 2a1 | 81 | 14 | 9.473 | 0.800 | 0.956 | 1.000 | -1948.179 | 4.604 |
| 1a7 | 81 | 8 | 7.508 | 0.483 | 0.957 | 1.000 | -1946.143 | 6.639 |
| 1a3 | 81 | 16 | 15.582 | 0.483 | 0.932 | 1.000 | -1946.070 | 6.712 |
| 1a1 | 81 | 11 | 11.654 | 0.390 | 0.947 | 0.981 | -1939.997 | 12.785 |
| 2a2 | 81 | 12 | 7.663 | 0.811 | 0.977 | 1.000 | -1933.989 | 18.794 |
| 1a6 | 81 | 14 | 12.089 | 0.599 | 0.966 | 1.000 | -1933.563 | 19.219 |
| 1a4 | 81 | 14 | 13.257 | 0.506 | 0.963 | 1.000 | -1932.395 | 20.387 |
| 2a3 | 81 | 15 | 47.565 | 0.000 | 0.835 | 0.063 | -1912.087 |  |
| 1a2 | 81 | 13 | 53.436 | 0.000 | 0.814 | 0.000 | -1902.215 |  |
| 1b7 | 81 | 17 | 20.875 | 0.232 | 0.911 | 0.905 | -1769.102 | 183.680 |
| 1b5 | 81 | 23 | 25.842 | 0.308 | 0.910 | 0.932 | -1768.135 | 184.647 |
| 2b5 | 81 | 21 | 22.822 | 0.354 | 0.917 | 0.957 | -1767.155 | 185.627 |
| 2b4 | 81 | 21 | 24.396 | 0.274 | 0.911 | 0.919 | -1765.582 | 187.201 |
| 1b3 | 81 | 23 | 28.549 | 0.196 | 0.901 | 0.868 | -1765.428 |  |
| 1b1 | 81 | 20 | 25.021 | 0.201 | 0.912 | 0.880 | -1762.957 |  |
| 2b1 | 81 | 17 | 20.399 | 0.254 | 0.923 | 0.919 | -1761.579 | 191.204 |
| 1b11 | 81 | 11 | 18.819 | 0.064 | 0.919 | 0.809 | -1759.159 |  |
| 2b2 | 81 | 15 | 18.935 | 0.217 | 0.932 | 0.906 | -1759.042 | 193.740 |
| 1b8 | 81 | 14 | 22.965 | 0.061 | 0.918 | 0.787 | -1753.013 |  |
| 1b6 | 81 | 21 | 25.649 | 0.220 | 0.939 | 0.889 | -1752.328 |  |
| 1b4 | 81 | 21 | 26.224 | 0.198 | 0.937 | 0.876 | -1751.753 |  |
| 1b10 | 81 | 18 | 23.923 | 0.158 | 0.943 | 0.859 | -1748.054 |  |
| 1b9 | 81 | 18 | 25.750 | 0.106 | 0.939 | 0.816 | -1746.227 |  |
| 2b3 | 81 | 18 | 57.588 | 0.000 | 0.828 | 0.058 | -1726.389 |  |
| 1b2 | 81 | 22 | 66.141 | 0.000 | 0.812 | 0.000 | -1725.837 |  |
| 1b7x | 80 | 17 | 22.472 | 0.167 | 0.901 | 0.837 | -1685.984 |  |
| 1b3x | 80 | 23 | 28.543 | 0.196 | 0.897 | 0.840 | -1683.913 |  |
| 2b4x | 80 | 21 | 25.418 | 0.230 | 0.905 | 0.872 | -1683.038 |  |
| 1b1x | 80 | 20 | 26.088 | 0.163 | 0.905 | 0.824 | -1680.368 |  |
| 2b1x | 80 | 17 | 22.510 | 0.166 | 0.914 | 0.841 | -1677.946 |  |
| 2b2x | 80 | 15 | 20.625 | 0.149 | 0.922 | 0.838 | -1675.832 |  |
| 1b8x | 80 | 14 | 20.080 | 0.128 | 0.926 | 0.824 | -1674.377 |  |
| 1b5x | 80 | 23 | 41.504 | 0.010 | 0.856 | 0.466 | -1670.952 |  |
| 2b5x | 80 | 21 | 38.296 | 0.012 | 0.860 | 0.501 | -1670.161 |  |
| 1b9x | 80 | 18 | 23.079 | 0.188 | 0.944 | 0.853 | -1667.377 |  |
| 1b2x | 80 | 22 | 43.411 | 0.004 | 0.855 | 0.382 | -1667.045 |  |
| 2b3x | 80 | 18 | 36.893 | 0.005 | 0.869 | 0.454 | -1665.563 |  |
| 1b6x | 80 | 21 | 34.862 | 0.029 | 0.916 | 0.600 | -1661.594 |  |
| 1b10x | 80 | 18 | 33.420 | 0.015 | 0.919 | 0.555 | -1657.037 |  |
| 1b4x | 80 | 30 | 65.565 | 0.000 | 0.878 | 0.597 | -1623.945 |  |
| 1b11x | 80 | 14 | 1119.186 | 0.000 | 0.699 | 0.028 | -1509.993 |  |
| *Primary 3 Asymmetry: Sylvia atricapilla* | | | | | | | | |
| **Model name^1^** | **n** | **dF** | **Chisq** | **P-value** | **GFI** | **CFI** | **AIC** | **ΔAIC** |
| 1a5 | 54 | 16 | 13.311 | 0.650 | 0.914 | 1.000 | -1397.490 | 0.000 |
| 1a7 | 54 | 8 | 5.803 | 0.669 | 0.951 | 1.000 | -1396.999 | 0.492 |
| 1a1 | 54 | 11 | 9.011 | 0.621 | 0.941 | 1.000 | -1391.791 | 5.700 |
| 1a3 | 54 | 16 | 19.903 | 0.225 | 0.873 | 0.838 | -1390.898 |  |
| 2a4 | 54 | 17 | 27.136 | 0.056 | 0.838 | 0.579 | -1385.665 |  |
| 2a5 | 54 | 17 | 27.227 | 0.055 | 0.847 | 0.575 | -1385.574 |  |
| 1a6 | 54 | 14 | 10.589 | 0.718 | 0.955 | 1.000 | -1384.213 | 13.277 |
| 2a1 | 54 | 14 | 24.504 | 0.040 | 0.861 | 0.564 | -1382.297 |  |
| 1a4 | 54 | 14 | 17.345 | 0.238 | 0.931 | 0.861 | -1377.456 |  |
| 1a2 | 54 | 13 | 29.226 | 0.006 | 0.844 | 0.326 | -1375.576 |  |
| 2a2 | 54 | 12 | 19.002 | 0.088 | 0.928 | 0.709 | -1371.799 |  |
| 2a3 | 54 | 15 | 39.495 | 0.001 | 0.813 | 0.000 | -1369.306 |  |
| 1b11 | 54 | 11 | 14.981 | 0.183 | 0.909 | 0.859 | -1247.312 |  |
| 1b7 | 54 | 17 | 29.943 | 0.027 | 0.835 | 0.543 | -1244.350 |  |
| 1b3 | 54 | 23 | 35.827 | 0.043 | 0.831 | 0.552 | -1242.466 |  |
| 1b8 | 54 | 14 | 18.189 | 0.198 | 0.908 | 0.854 | -1242.104 |  |
| 1b5 | 54 | 23 | 36.529 | 0.036 | 0.833 | 0.527 | -1241.764 |  |
| 1b1 | 54 | 20 | 33.151 | 0.032 | 0.845 | 0.540 | -1239.142 |  |
| 2b4 | 54 | 21 | 36.671 | 0.018 | 0.826 | 0.452 | -1237.622 |  |
| 2b5 | 54 | 21 | 37.277 | 0.016 | 0.827 | 0.431 | -1237.016 |  |
| 1b10 | 54 | 18 | 20.370 | 0.312 | 0.929 | 0.917 | -1235.923 | 161.567 |
| 2b2 | 54 | 15 | 28.393 | 0.019 | 0.865 | 0.532 | -1233.900 |  |
| 2b1 | 54 | 17 | 33.888 | 0.009 | 0.840 | 0.410 | -1232.405 |  |
| 1b9 | 54 | 18 | 27.264 | 0.074 | 0.907 | 0.676 | -1229.029 |  |
| 1b4 | 54 | 21 | 33.269 | 0.043 | 0.891 | 0.571 | -1229.024 |  |
| 1b6 | 54 | 21 | 33.806 | 0.038 | 0.889 | 0.552 | -1228.487 |  |
| 1b2 | 54 | 22 | 48.415 | 0.001 | 0.804 | 0.077 | -1227.878 |  |
| 2b3 | 54 | 18 | 48.729 | 0.000 | 0.801 | 0.000 | -1219.563 |  |
| 1b7x | 53 | 17 | 30.195 | 0.025 | 0.831 | 0.141 | -1160.100 |  |
| 1b3x | 53 | 23 | 35.753 | 0.044 | 0.826 | 0.183 | -1158.542 |  |
| 1b5x | 53 | 23 | 36.086 | 0.040 | 0.835 | 0.161 | -1158.209 |  |
| 1b2x | 53 | 22 | 36.660 | 0.026 | 0.832 | 0.061 | -1155.635 |  |
| 2b4x | 53 | 21 | 35.079 | 0.028 | 0.829 | 0.098 | -1155.216 |  |
| 1b1x | 53 | 20 | 33.512 | 0.030 | 0.840 | 0.134 | -1154.783 |  |
| 1b8x | 53 | 14 | 21.667 | 0.086 | 0.891 | 0.509 | -1154.629 |  |
| 2b5x | 53 | 21 | 36.515 | 0.019 | 0.833 | 0.006 | -1153.780 |  |
| 2b2x | 53 | 15 | 27.422 | 0.025 | 0.868 | 0.204 | -1150.874 |  |
| 2b1x | 53 | 17 | 32.728 | 0.012 | 0.842 | 0.000 | -1149.567 |  |
| 1b10x | 53 | 18 | 23.035 | 0.189 | 0.918 | 0.677 | -1149.260 |  |
| 2b3x | 53 | 18 | 35.643 | 0.008 | 0.835 | 0.000 | -1148.652 |  |
| 1b4x | 53 | 30 | 64.455 | 0.000 | 0.831 | 0.318 | -1146.479 |  |
| 1b6x | 53 | 21 | 34.421 | 0.033 | 0.884 | 0.140 | -1143.874 |  |
| 1b9x | 53 | 18 | 31.579 | 0.025 | 0.893 | 0.130 | -1140.717 |  |
| 1b11x | 53 | 14 | 749.663 | 0.000 | 0.692 | 0.022 | -1032.295 |  |
| *Primary 3 Asymmetry: Turdus merula* | | | | | | | | |
| **Model name^1^** | **n** | **dF** | **Chisq** | **P-value** | **GFI** | **CFI** | **AIC** | **ΔAIC** |
| 2a4 | 50 | 17 | 12.919 | 0.742 | 0.910 | 1.000 | -1289.449 | 0.000 |
| 2a3 | 50 | 15 | 10.180 | 0.808 | 0.929 | 1.000 | -1288.188 | 1.261 |
| 2a5 | 50 | 17 | 14.453 | 0.635 | 0.895 | 1.000 | -1287.915 |  |
| 2a1 | 50 | 14 | 8.533 | 0.860 | 0.938 | 1.000 | -1287.835 | 1.614 |
| 1a3 | 50 | 16 | 12.581 | 0.703 | 0.911 | 1.000 | -1287.787 | 1.661 |
| 1a5 | 50 | 16 | 16.162 | 0.442 | 0.889 | 0.000 | -1284.206 |  |
| 1a7 | 50 | 8 | 8.786 | 0.361 | 0.922 | 0.000 | -1283.582 |  |
| 1a2 | 50 | 13 | 13.665 | 0.398 | 0.901 | 0.000 | -1280.703 |  |
| 1a1 | 50 | 11 | 10.274 | 0.506 | 0.928 | 1.000 | -1280.094 | 9.354 |
| 1a4 | 50 | 14 | 11.104 | 0.678 | 0.950 | 1.000 | -1273.264 | 16.184 |
| 2a2 | 50 | 12 | 7.538 | 0.820 | 0.965 | 1.000 | -1272.830 | 16.619 |
| 1a6 | 50 | 14 | 11.891 | 0.615 | 0.946 | 1.000 | -1272.477 | 16.972 |
| 1b3 | 50 | 23 | 26.525 | 0.277 | 0.860 | 0.553 | -1168.196 |  |
| 1b7 | 50 | 17 | 22.713 | 0.159 | 0.855 | 0.391 | -1168.009 |  |
| 1b2 | 50 | 22 | 26.561 | 0.228 | 0.861 | 0.421 | -1166.160 |  |
| 2b4 | 50 | 21 | 25.236 | 0.237 | 0.863 | 0.462 | -1165.485 |  |
| 1b1 | 50 | 20 | 24.201 | 0.234 | 0.870 | 0.467 | -1164.520 |  |
| 1b5 | 50 | 23 | 31.329 | 0.115 | 0.828 | 0.000 | -1163.393 |  |
| 2b3 | 50 | 18 | 22.031 | 0.231 | 0.877 | 0.488 | -1162.690 |  |
| 2b5 | 50 | 21 | 28.530 | 0.126 | 0.834 | 0.044 | -1162.191 |  |
| 2b1 | 50 | 17 | 20.579 | 0.246 | 0.883 | 0.546 | -1162.142 |  |
| 2b2 | 50 | 15 | 18.487 | 0.238 | 0.899 | 0.558 | -1160.235 |  |
| 1b11 | 50 | 11 | 19.158 | 0.058 | 0.878 | 0.131 | -1159.563 |  |
| 1b8 | 50 | 14 | 20.646 | 0.111 | 0.889 | 0.157 | -1156.075 |  |
| 1b4 | 50 | 21 | 25.048 | 0.245 | 0.904 | 0.486 | -1153.673 |  |
| 1b6 | 50 | 21 | 27.058 | 0.169 | 0.899 | 0.231 | -1151.664 |  |
| 1b9 | 50 | 18 | 22.925 | 0.193 | 0.912 | 0.375 | -1149.797 |  |
| 1b10 | 50 | 18 | 25.344 | 0.116 | 0.905 | 0.068 | -1147.377 |  |
| 1b4x | 49 | 30 | 50.687 | 0.011 | 0.847 | 0.000 | -1123.779 |  |
| 1b3x | 49 | 23 | 25.078 | 0.346 | 0.864 | 0.000 | -1085.927 |  |
| 1b7x | 49 | 17 | 21.594 | 0.201 | 0.861 | 0.000 | -1085.412 |  |
| 1b2x | 49 | 22 | 24.275 | 0.333 | 0.871 | 0.000 | -1084.731 |  |
| 1b5x | 49 | 23 | 26.356 | 0.284 | 0.858 | 0.000 | -1084.650 |  |
| 2b5x | 49 | 21 | 23.030 | 0.342 | 0.870 | 0.000 | -1083.975 |  |
| 2b4x | 49 | 21 | 24.017 | 0.292 | 0.866 | 0.000 | -1082.989 |  |
| 1b1x | 49 | 20 | 23.575 | 0.261 | 0.871 | 0.000 | -1081.431 |  |
| 2b3x | 49 | 18 | 20.336 | 0.314 | 0.881 | 0.000 | -1080.670 |  |
| 2b1x | 49 | 17 | 20.138 | 0.267 | 0.883 | 0.000 | -1078.867 |  |
| 2b2x | 49 | 15 | 18.354 | 0.244 | 0.899 | 0.000 | -1076.652 |  |
| 1b8x | 49 | 14 | 21.411 | 0.092 | 0.883 | 0.000 | -1071.595 |  |
| 1b6x | 49 | 21 | 24.454 | 0.272 | 0.908 | 0.000 | -1070.552 |  |
| 1b10x | 49 | 18 | 22.880 | 0.195 | 0.914 | 0.000 | -1066.126 |  |
| 1b9x | 49 | 18 | 23.615 | 0.168 | 0.908 | 0.000 | -1065.390 |  |
| 1b11x | 49 | 14 | 651.374 | 0.000 | 0.689 | 0.000 | -970.314 |  |
| *Primary 3 Asymmetry: Turdus philomelos* | | | | | | | | |
| **Model name^1^** | **n** | **dF** | **Chisq** | **P-value** | **GFI** | **CFI** | **AIC** | **ΔAIC** |
| 1a3 | 35 | 16 | 28.616 | 0.027 | 0.790 | 0.386 | -937.584 |  |
| 2a4 | 35 | 17 | 30.651 | 0.022 | 0.779 | 0.336 | -937.549 |  |
| 2a5 | 35 | 17 | 35.076 | 0.006 | 0.763 | 0.121 | -933.125 |  |
| 2a1 | 35 | 14 | 29.471 | 0.009 | 0.787 | 0.248 | -932.729 |  |
| 1a7 | 35 | 8 | 26.622 | 0.001 | 0.762 | 0.198 | -931.578 |  |
| 1a5 | 35 | 16 | 34.993 | 0.004 | 0.761 | 0.076 | -931.207 |  |
| 2a3 | 35 | 15 | 33.772 | 0.004 | 0.764 | 0.087 | -930.428 |  |
| 1a1 | 35 | 11 | 27.960 | 0.003 | 0.793 | 0.175 | -928.241 |  |
| 1a2 | 35 | 13 | 36.257 | 0.001 | 0.746 | 0.000 | -923.943 |  |
| 1a4 | 35 | 14 | 28.207 | 0.013 | 0.859 | 0.309 | -921.994 |  |
| 1a6 | 35 | 14 | 29.942 | 0.008 | 0.851 | 0.225 | -920.258 |  |
| 2a2 | 35 | 12 | 26.599 | 0.009 | 0.866 | 0.290 | -919.601 |  |
| 1b3 | 35 | 23 | 41.006 | 0.012 | 0.766 | 0.341 | -862.974 |  |
| 1b7 | 35 | 17 | 38.906 | 0.002 | 0.743 | 0.269 | -861.073 |  |
| 2b4 | 35 | 21 | 40.312 | 0.007 | 0.767 | 0.293 | -859.667 |  |
| 1b5 | 35 | 23 | 46.131 | 0.003 | 0.744 | 0.153 | -857.849 |  |
| 1b1 | 35 | 20 | 40.244 | 0.005 | 0.771 | 0.259 | -857.735 |  |
| 2b5 | 35 | 21 | 45.547 | 0.001 | 0.748 | 0.102 | -854.432 |  |
| 1b2 | 35 | 22 | 47.795 | 0.001 | 0.728 | 0.056 | -854.184 |  |
| 2b1 | 35 | 17 | 38.890 | 0.002 | 0.776 | 0.199 | -853.089 |  |
| 1b11 | 35 | 11 | 35.174 | 0.000 | 0.766 | 0.194 | -852.805 |  |
| 2b2 | 35 | 15 | 36.154 | 0.002 | 0.790 | 0.226 | -851.826 |  |
| 2b3 | 35 | 18 | 42.973 | 0.001 | 0.757 | 0.086 | -851.006 |  |
| 1b8 | 35 | 14 | 36.512 | 0.001 | 0.790 | 0.176 | -849.467 |  |
| 1b4 | 35 | 21 | 40.596 | 0.006 | 0.833 | 0.283 | -847.383 |  |
| 1b6 | 35 | 21 | 41.080 | 0.005 | 0.833 | 0.265 | -846.900 |  |
| 1b9 | 35 | 18 | 37.188 | 0.005 | 0.845 | 0.298 | -844.791 |  |
| 1b10 | 35 | 18 | 40.392 | 0.002 | 0.835 | 0.180 | -841.588 |  |
| 1b3x | 35 | 23 | 36.059 | 0.041 | 0.791 | 0.507 | -833.180 |  |
| 1b7x | 35 | 17 | 33.959 | 0.009 | 0.768 | 0.419 | -831.281 |  |
| 1b1x | 35 | 20 | 34.109 | 0.025 | 0.799 | 0.468 | -829.130 |  |
| 2b4x | 35 | 21 | 36.768 | 0.018 | 0.784 | 0.405 | -828.471 |  |
| 1b2x | 35 | 22 | 40.860 | 0.009 | 0.757 | 0.289 | -826.380 |  |
| 2b3x | 35 | 18 | 35.053 | 0.009 | 0.793 | 0.357 | -824.186 |  |
| 2b1x | 35 | 17 | 34.157 | 0.008 | 0.797 | 0.353 | -823.082 |  |
| 1b4x | 35 | 30 | 70.302 | 0.000 | 0.781 | 0.281 | -822.390 |  |
| 1b5x | 35 | 23 | 47.775 | 0.002 | 0.736 | 0.065 | -821.464 |  |
| 2b2x | 35 | 15 | 33.146 | 0.004 | 0.804 | 0.315 | -820.093 |  |
| 1b8x | 35 | 14 | 33.285 | 0.003 | 0.806 | 0.273 | -817.954 |  |
| 2b5x | 35 | 21 | 47.923 | 0.001 | 0.734 | 0.000 | -817.316 |  |
| 1b6x | 35 | 21 | 36.215 | 0.021 | 0.849 | 0.426 | -817.024 |  |
| 1b9x | 35 | 18 | 33.961 | 0.013 | 0.858 | 0.398 | -813.279 |  |
| 1b10x | 35 | 18 | 35.759 | 0.008 | 0.851 | 0.330 | -811.480 |  |
| 1b11x | 35 | 14 | 316.767 | 0.000 | 0.636 | 0.049 | -758.387 |  |

**^1^ Model name:** The first number refers to whether the model is amended with a measure for vector abundance (1: original model, without measure for vector abundance, 2: model including measure for vector abundance). The first letter describes the underlying hypothesis (a: 'top-down'-hypothesis, b: 'bottom-up'-hypothesis, see Background). The number following the letter indicates the model number (not further described). The addition of an 'x' indicated whether that model was modelled using the species-specific parasite-vector relationship (i.e. *Plasmodium/Haemoproteus* infection in relation the abundance of vectors of the families Culicidae/Ceratopogonidae). E.g.: 1b11x indicates the original model (1), model number (11), 'bottom-up'-hypothesis (addition of 'x').

**Table S4.** Summary of the best-fitted structural equation models (P>0.05, GFI>0.9, CFI>0.9 and lowest AIC compared to other models that met these criteria), also including models with ΔAIC<2 are indicated (grey shaded). GFI = goodness-of-fit index; CFI = comparative fit index or Bentler comparative fit index; AIC = Akaike Information Criterion. For the explanation of the model names, see footnote.

|  | Tarsus Asymmetry | | | | | | | | |
| --- | --- | --- | --- | --- | --- | --- | --- | --- | --- |
| **Species** | **Model name^1^** | **n** | **dF** | **Chisq** | **P-value** | **GFI** | **CFI** | **AIC** | **ΔAIC** |
| all | 1a7 | 387 | 8 | 4.75 | 0.78 | 0.99 | 1.00 | -7889.14 | 0.00 |
| *Cyanistes caeruleus* | - | - | - | - | - | - | - | - | - |
| *Erithacus rubecula* | - | - | - | - | - | - | - | - | - |
| *Fringilla coelebs* | - | - | - | - | - | - | - | - | - |
| *Parus major* | 2a5 | 84 | 17 | 14.57 | 0.63 | 0.94 | 1.00 | -1801.67 | 0.00 |
| *Parus major* | 2a4 | 84 | 17 | 14.99 | 0.60 | 0.94 | 1.00 | -1801.25 | 0.42 |
| *Parus major* | 1a5 | 84 | 16 | 13.06 | 0.67 | 0.94 | 1.00 | -1801.18 | 0.49 |
| *Parus major* | 2a1 | 84 | 14 | 10.18 | 0.75 | 0.96 | 1.00 | -1800.06 | 1.61 |
| *Sylvia atricapilla* | 1a6 | 61 | 14 | 16.42 | 0.29 | 0.94 | 0.92 | -1382.10 | 0.00 |
| *Turdus merula* | 1a3 | 58 | 16 | 8.29 | 0.94 | 0.95 | 1.00 | -1346.26 | 0.00 |
| *Turdus philomelos* | - | - | - | - | - | - | - | - | - |

|  | | Primary 3 Asymmetry | | | | | | | | |
| --- | --- | --- | --- | --- | --- | --- | --- | --- | --- | --- |
| **Species** | **Model name^1^** | | **n** | **dF** | **Chisq** | **P-value** | **GFI** | **CFI** | **AIC** | **ΔAIC** |
| all | 1a7 | | 360 | 8 | 3.05 | 0.93 | 1.00 | 1.00 | -8197.55 | 0.00 |
| *Cyanistes caeruleus* | - | | - | - | - | - | - | - | - | - |
| *Erithacus rubecula* | 1a7 | | 68 | 8 | 11.92 | 0.15 | 0.92 | 0.93 | -1708.28 | 0.00 |
| *Fringilla coelebs* | 1a7 | | 41 | 8 | 7.87 | 0.45 | 0.92 | 1.00 | -925.10 | 0.00 |
| *Parus major* | 2a5 | | 81 | 17 | 10.87 | 0.86 | 0.95 | 1.00 | -1952.78 | 0.00 |
| *Parus major* | 2a4 | | 81 | 17 | 12.02 | 0.80 | 0.94 | 1.00 | -1951.63 | 1.15 |
| *Sylvia atricapilla* | 1a5 | | 54 | 16 | 13.31 | 0.65 | 0.91 | 1.00 | -1397.49 | 0.00 |
| *Sylvia atricapilla* | 1a7 | | 54 | 8 | 5.80 | 0.67 | 0.95 | 1.00 | -1397.00 | 0.49 |
| *Turdus merula* | 2a4 | | 50 | 17 | 12.92 | 0.74 | 0.91 | 1.00 | -1289.45 | 0.00 |
| *Turdus merula* | 2a3 | | 50 | 15 | 10.18 | 0.81 | 0.93 | 1.00 | -1288.19 | 1.26 |
| *Turdus philomelos* | - | | - | - | - | - | - | - | - | - |

**^1^ Model name:** The first number refers to whether the model is amended with a measure for vector abundance (1: original model, without measure for vector abundance, 2: model including measure for vector abundance). The first letter describes the underlying hypothesis (a: 'top-down'-hypothesis, b: 'bottom-up'-hypothesis, see Background). The number following the letter indicates the model number (not further described). The addition of an 'x' indicated whether that model was modelled using the species-specific parasite-vector relationship (i.e. *Plasmodium/Haemoproteus* infection in relation the abundance of vectors of the families Culicidae/Ceratopogonidae). E.g.: 1b11x indicates the original model (1), model number (11), 'bottom-up'-hypothesis (addition of 'x').

**Table S5.** Detailed SEM results for the best fitted structural equation models (SEM) for both tarsus and primary asymmetry, significant pathways are in grey fields and in bold. For the explanation of the model names, see footnote.

| ***Overall, tarsus asymmetry (hypothesis 1a, model name 1a7); Fig. 2*** | | | | | | | | |
| --- | --- | --- | --- | --- | --- | --- | --- | --- |
| **Pathway** | | | **Estimate** | **SE** | **z** | **P-value** | **CI, lower** | **CI, upper** |
| m_pcr_infected | ~ | So1 | -1.00 | 2.06 | -0.49 | 0.63 | -5.03 | 3.03 |
| m_pcr_infected | ~ | Ga | 0.70 | 2.02 | 0.35 | 0.73 | -3.26 | 4.66 |
| m_pcr_infected | ~ | En | 1.43 | 1.19 | 1.20 | 0.23 | -0.90 | 3.77 |
| m_pcr_parasite_count | ~ | So1 | -8.37 | 3.36 | -2.49 | 0.01 | -14.95 | -1.79 |
| m_pcr_parasite_count | ~ | Ga | 6.85 | 3.30 | 2.08 | 0.04 | 0.38 | 13.33 |
| m_pcr_parasite_count | ~ | En | 0.46 | 1.95 | 0.24 | 0.81 | -3.35 | 4.28 |
| m_HL_ratio_0ifna | ~ | m_pcr_infected | 0.04 | 0.03 | 1.52 | 0.13 | -0.01 | 0.10 |
| m_HL_ratio_0ifna | ~ | m_pcr_parasite_count | 0.01 | 0.02 | 0.58 | 0.56 | -0.02 | 0.04 |
| m_HL_ratio_0ifna | ~ | tarsus_asymmetry | 2.00 | 1.28 | 1.56 | 0.12 | -0.51 | 4.51 |
| m_leucocytes_0ifna | ~ | m_pcr_infected | 0.25 | 0.09 | 2.89 | 0.00 | 0.08 | 0.43 |
| m_leucocytes_0ifna | ~ | m_pcr_parasite_count | -0.24 | 0.05 | -4.49 | 0.00 | -0.35 | -0.14 |
| m_leucocytes_0ifna | ~ | tarsus_asymmetry | 7.73 | 4.08 | 1.90 | 0.06 | -0.27 | 15.73 |
| m_pcr_infected | ~~ | m_pcr_parasite_count | 0.16 | 0.02 | 9.89 | 0.00 | 0.12 | 0.19 |
| m_HL_ratio_0ifna | ~~ | m_leucocytes_0ifna | 0.00 | 0.01 | -0.66 | 0.51 | -0.01 | 0.01 |
| m_pcr_infected | ~~ | m_pcr_infected | 0.16 | 0.01 | 13.91 | 0.00 | 0.14 | 0.19 |
| m_pcr_parasite_count | ~~ | m_pcr_parasite_count | 0.44 | 0.03 | 13.91 | 0.00 | 0.37 | 0.50 |
| m_HL_ratio_0ifna | ~~ | m_HL_ratio_0ifna | 0.03 | 0.00 | 13.91 | 0.00 | 0.03 | 0.04 |
| m_leucocytes_0ifna | ~~ | m_leucocytes_0ifna | 0.33 | 0.02 | 13.91 | 0.00 | 0.28 | 0.37 |
| So1 | ~~ | So1 | 0.00 | 0.00 | NA | NA | 0.00 | 0.00 |
| So1 | ~~ | Ga | 0.00 | 0.00 | NA | NA | 0.00 | 0.00 |
| So1 | ~~ | En | 0.00 | 0.00 | NA | NA | 0.00 | 0.00 |
| So1 | ~~ | tarsus_asymmetry | 0.00 | 0.00 | NA | NA | 0.00 | 0.00 |
| Ga | ~~ | Ga | 0.00 | 0.00 | NA | NA | 0.00 | 0.00 |
| Ga | ~~ | En | 0.00 | 0.00 | NA | NA | 0.00 | 0.00 |
| Ga | ~~ | tarsus_asymmetry | 0.00 | 0.00 | NA | NA | 0.00 | 0.00 |
| En | ~~ | En | 0.00 | 0.00 | NA | NA | 0.00 | 0.00 |
| En | ~~ | tarsus_asymmetry | 0.00 | 0.00 | NA | NA | 0.00 | 0.00 |
| tarsus_asymmetry | ~~ | tarsus_asymmetry | 0.00 | 0.00 | NA | NA | 0.00 | 0.00 |
|  |  |  |  |  |  |  |  |  |
| ***P. major, tarsus asymmetry (hypothesis 2a, model name 2a5); Fig. S3a*** | | | | | | | | |
| **Pathway** | | | **Estimate** | **SE** | **z** | **P-value** | **CI, lower** | **CI, upper** |
| tarsus_asymmetry | ~ | So1 | 0.05 | 0.09 | 0.48 | 0.63 | -0.14 | 0.23 |
| tarsus_asymmetry | ~ | Ga | -0.08 | 0.10 | -0.85 | 0.40 | -0.27 | 0.11 |
| tarsus_asymmetry | ~ | En | 0.01 | 0.05 | 0.27 | 0.78 | -0.09 | 0.12 |
| m_pcr_parasite_count | ~ | tarsus_asymmetry | -2.47 | 8.10 | -0.31 | 0.76 | -18.35 | 13.41 |
| m_HL_ratio_0ifna | ~ | m_pcr_parasite_count | -0.05 | 0.03 | -1.64 | 0.10 | -0.11 | 0.01 |
| m_leucocytes_0ifna | ~ | m_pcr_parasite_count | -0.06 | 0.08 | -0.77 | 0.44 | -0.22 | 0.10 |
| m_pcr_parasite_count | ~~ | m_pcr_infected | 0.14 | 0.03 | 4.67 | 0.00 | 0.08 | 0.20 |
| m_HL_ratio_0ifna | ~~ | m_leucocytes_0ifna | 0.02 | 0.01 | 1.77 | 0.08 | 0.00 | 0.04 |
| tarsus_asymmetry | ~~ | tarsus_asymmetry | 0.00 | 0.00 | 6.48 | 0.00 | 0.00 | 0.00 |
| m_pcr_parasite_count | ~~ | m_pcr_parasite_count | 0.44 | 0.07 | 6.48 | 0.00 | 0.31 | 0.58 |
| m_HL_ratio_0ifna | ~~ | m_HL_ratio_0ifna | 0.03 | 0.01 | 6.48 | 0.00 | 0.02 | 0.04 |
| m_leucocytes_0ifna | ~~ | m_leucocytes_0ifna | 0.25 | 0.04 | 6.48 | 0.00 | 0.17 | 0.32 |
| m_pcr_infected | ~~ | m_pcr_infected | 0.13 | 0.02 | 6.48 | 0.00 | 0.09 | 0.17 |
| So1 | ~~ | So1 | 0.00 | 0.00 | NA | NA | 0.00 | 0.00 |
| So1 | ~~ | Ga | 0.00 | 0.00 | NA | NA | 0.00 | 0.00 |
| So1 | ~~ | En | 0.00 | 0.00 | NA | NA | 0.00 | 0.00 |
| Ga | ~~ | Ga | 0.00 | 0.00 | NA | NA | 0.00 | 0.00 |
| Ga | ~~ | En | 0.00 | 0.00 | NA | NA | 0.00 | 0.00 |
| En | ~~ | En | 0.00 | 0.00 | NA | NA | 0.00 | 0.00 |
|  |  |  |  |  |  |  |  |  |
| ***S. atricapilla, tarsus asymmetry (hypothesis 1a, model name 1a6); Fig. S3b*** | | | | | | | | |
| **Pathway** | | | **Estimate** | **SE** | **z** | **P-value** | **CI, lower** | **CI, upper** |
| m_pcr_parasite_count | ~ | So1 | -13.41 | 8.01 | -1.67 | 0.09 | -29.11 | 2.30 |
| m_pcr_parasite_count | ~ | Ga | 19.81 | 5.24 | 3.78 | 0.00 | 9.54 | 30.08 |
| m_pcr_parasite_count | ~ | En | -5.55 | 3.48 | -1.59 | 0.11 | -12.38 | 1.28 |
| m_HL_ratio_0ifna | ~ | m_pcr_infected | 0.16 | 0.11 | 1.50 | 0.13 | -0.05 | 0.38 |
| m_HL_ratio_0ifna | ~ | m_pcr_parasite_count | -0.05 | 0.05 | -1.07 | 0.28 | -0.14 | 0.04 |
| m_leucocytes_0ifna | ~ | m_pcr_infected | 0.15 | 0.23 | 0.65 | 0.52 | -0.30 | 0.59 |
| m_leucocytes_0ifna | ~ | m_pcr_parasite_count | 0.09 | 0.10 | 0.95 | 0.35 | -0.10 | 0.29 |
| tarsus_asymmetry | ~ | m_HL_ratio_0ifna | 0.01 | 0.01 | 0.82 | 0.41 | -0.01 | 0.02 |
| tarsus_asymmetry | ~ | m_leucocytes_0ifna | 0.00 | 0.00 | -0.56 | 0.58 | -0.01 | 0.00 |
| m_pcr_parasite_count | ~~ | m_pcr_infected | 0.07 | 0.02 | 3.75 | 0.00 | 0.03 | 0.11 |
| m_HL_ratio_0ifna | ~~ | m_leucocytes_0ifna | 0.00 | 0.01 | -0.48 | 0.63 | -0.02 | 0.01 |
| m_pcr_parasite_count | ~~ | m_pcr_parasite_count | 0.26 | 0.05 | 5.52 | 0.00 | 0.17 | 0.36 |
| m_HL_ratio_0ifna | ~~ | m_HL_ratio_0ifna | 0.03 | 0.01 | 5.52 | 0.00 | 0.02 | 0.05 |
| m_leucocytes_0ifna | ~~ | m_leucocytes_0ifna | 0.15 | 0.03 | 5.52 | 0.00 | 0.09 | 0.20 |
| tarsus_asymmetry | ~~ | tarsus_asymmetry | 0.00 | 0.00 | 5.52 | 0.00 | 0.00 | 0.00 |
| m_pcr_infected | ~~ | m_pcr_infected | 0.06 | 0.01 | 5.52 | 0.00 | 0.04 | 0.08 |
| So1 | ~~ | So1 | 0.00 | 0.00 | 5.52 | 0.00 | 0.00 | 0.00 |
| So1 | ~~ | Ga | 0.00 | 0.00 | 3.86 | 0.00 | 0.00 | 0.00 |
| So1 | ~~ | En | 0.00 | 0.00 | -3.49 | 0.00 | 0.00 | 0.00 |
| Ga | ~~ | Ga | 0.00 | 0.00 | 5.52 | 0.00 | 0.00 | 0.00 |
| Ga | ~~ | En | 0.00 | 0.00 | -2.51 | 0.01 | 0.00 | 0.00 |
| En | ~~ | En | 0.00 | 0.00 | 5.52 | 0.00 | 0.00 | 0.00 |
|  |  |  |  |  |  |  |  |  |
| ***T. merula, tarsus asymmetry (hypothesis 1a, model name 1a3); Fig. S3c*** | | | | | | | | |
| **Pathway** | | | **Estimate** | **SE** | **z** | **P-value** | **CI, lower** | **CI, upper** |
| m_pcr_infected | ~ | So1 | -1.85 | 1.37 | -1.35 | 0.18 | -4.53 | 0.84 |
| m_pcr_infected | ~ | Ga | 1.19 | 1.48 | 0.80 | 0.42 | -1.72 | 4.10 |
| m_pcr_infected | ~ | En | -0.67 | 0.93 | -0.72 | 0.48 | -2.50 | 1.16 |
| m_HL_ratio_0ifna | ~ | m_pcr_infected | 0.15 | 0.19 | 0.78 | 0.44 | -0.23 | 0.52 |
| m_leucocytes_0ifna | ~ | m_pcr_infected | -0.61 | 0.32 | -1.91 | 0.06 | -1.23 | 0.02 |
| tarsus_asymmetry | ~ | m_HL_ratio_0ifna | 0.02 | 0.01 | 2.83 | 0.01 | 0.01 | 0.03 |
| tarsus_asymmetry | ~ | m_leucocytes_0ifna | 0.00 | 0.00 | 0.73 | 0.47 | 0.00 | 0.01 |
| m_pcr_infected | ~~ | m_pcr_parasite_count | 0.01 | 0.01 | 1.63 | 0.10 | 0.00 | 0.03 |
| m_HL_ratio_0ifna | ~~ | m_leucocytes_0ifna | -0.01 | 0.01 | -0.88 | 0.38 | -0.02 | 0.01 |
| m_pcr_infected | ~~ | m_pcr_infected | 0.02 | 0.00 | 5.39 | 0.00 | 0.01 | 0.02 |
| m_HL_ratio_0ifna | ~~ | m_HL_ratio_0ifna | 0.04 | 0.01 | 5.39 | 0.00 | 0.02 | 0.05 |
| m_leucocytes_0ifna | ~~ | m_leucocytes_0ifna | 0.10 | 0.02 | 5.39 | 0.00 | 0.06 | 0.14 |
| tarsus_asymmetry | ~~ | tarsus_asymmetry | 0.00 | 0.00 | 5.39 | 0.00 | 0.00 | 0.00 |
| m_pcr_parasite_count | ~~ | m_pcr_parasite_count | 0.22 | 0.04 | 5.39 | 0.00 | 0.14 | 0.31 |
| So1 | ~~ | So1 | 0.00 | 0.00 | NA | NA | 0.00 | 0.00 |
| So1 | ~~ | Ga | 0.00 | 0.00 | NA | NA | 0.00 | 0.00 |
| So1 | ~~ | En | 0.00 | 0.00 | NA | NA | 0.00 | 0.00 |
| Ga | ~~ | Ga | 0.00 | 0.00 | NA | NA | 0.00 | 0.00 |
| Ga | ~~ | En | 0.00 | 0.00 | NA | NA | 0.00 | 0.00 |
| En | ~~ | En | 0.00 | 0.00 | NA | NA | 0.00 | 0.00 |

| ***Overall, primary 3 asymmetry (hypothesis 1a, model name 1a7); Fig. S4a*** | | | | | | | | |
| --- | --- | --- | --- | --- | --- | --- | --- | --- |
| **Pathway** | | | **Estimate** | **SE** | **z** | **P-value** | **CI, lower** | **CI, upper** |
| m_pcr_infected | ~ | So1 | -1.11 | 2.11 | -0.53 | 0.60 | -5.24 | 3.03 |
| m_pcr_infected | ~ | Ga | -0.61 | 2.11 | -0.29 | 0.77 | -4.74 | 3.52 |
| m_pcr_infected | ~ | En | 0.82 | 1.24 | 0.67 | 0.51 | -1.60 | 3.25 |
| m_pcr_parasite_count | ~ | So1 | -9.80 | 3.50 | -2.80 | 0.01 | -16.67 | -2.94 |
| m_pcr_parasite_count | ~ | Ga | 6.02 | 3.50 | 1.72 | 0.09 | -0.85 | 12.88 |
| m_pcr_parasite_count | ~ | En | -0.27 | 2.05 | -0.13 | 0.90 | -4.30 | 3.76 |
| m_HL_ratio_0ifna | ~ | m_pcr_infected | 0.03 | 0.03 | 1.05 | 0.29 | -0.03 | 0.09 |
| m_HL_ratio_0ifna | ~ | m_pcr_parasite_count | 0.02 | 0.02 | 0.94 | 0.35 | -0.02 | 0.05 |
| m_HL_ratio_0ifna | ~ | p3_asymmetry | 4.40 | 4.39 | 1.00 | 0.32 | -4.21 | 13.01 |
| m_leucocytes_0ifna | ~ | m_pcr_infected | 0.26 | 0.09 | 2.79 | 0.01 | 0.08 | 0.43 |
| m_leucocytes_0ifna | ~ | m_pcr_parasite_count | -0.24 | 0.06 | -4.34 | 0.00 | -0.35 | -0.13 |
| m_leucocytes_0ifna | ~ | p3_asymmetry | 23.72 | 13.93 | 1.70 | 0.09 | -3.58 | 51.01 |
| m_pcr_infected | ~~ | m_pcr_parasite_count | 0.16 | 0.02 | 9.53 | 0.00 | 0.12 | 0.19 |
| m_HL_ratio_0ifna | ~~ | m_leucocytes_0ifna | 0.00 | 0.01 | -0.57 | 0.57 | -0.01 | 0.01 |
| m_pcr_infected | ~~ | m_pcr_infected | 0.16 | 0.01 | 13.42 | 0.00 | 0.14 | 0.19 |
| m_pcr_parasite_count | ~~ | m_pcr_parasite_count | 0.45 | 0.03 | 13.42 | 0.00 | 0.38 | 0.51 |
| m_HL_ratio_0ifna | ~~ | m_HL_ratio_0ifna | 0.03 | 0.00 | 13.42 | 0.00 | 0.03 | 0.04 |
| m_leucocytes_0ifna | ~~ | m_leucocytes_0ifna | 0.33 | 0.02 | 13.42 | 0.00 | 0.28 | 0.38 |
| So1 | ~~ | So1 | 0.00 | 0.00 | NA | NA | 0.00 | 0.00 |
| So1 | ~~ | Ga | 0.00 | 0.00 | NA | NA | 0.00 | 0.00 |
| So1 | ~~ | En | 0.00 | 0.00 | NA | NA | 0.00 | 0.00 |
| So1 | ~~ | p3_asymmetry | 0.00 | 0.00 | NA | NA | 0.00 | 0.00 |
| Ga | ~~ | Ga | 0.00 | 0.00 | NA | NA | 0.00 | 0.00 |
| Ga | ~~ | En | 0.00 | 0.00 | NA | NA | 0.00 | 0.00 |
| Ga | ~~ | p3_asymmetry | 0.00 | 0.00 | NA | NA | 0.00 | 0.00 |
| En | ~~ | En | 0.00 | 0.00 | NA | NA | 0.00 | 0.00 |
| En | ~~ | p3_asymmetry | 0.00 | 0.00 | NA | NA | 0.00 | 0.00 |
| p3_asymmetry | ~~ | p3_asymmetry | 0.00 | 0.00 | NA | NA | 0.00 | 0.00 |
|  |  |  |  |  |  |  |  |  |
| ***E. rubecula, primary 3 asymmetry (hypothesis 1a, model name 1a7); Fig. S4b*** | | | | | | | | |
| **Pathway** | | | **Estimate** | **SE** | **z** | **P-value** | **CI, lower** | **CI, upper** |
| m_pcr_infected | ~ | So1 | -5.59 | 5.58 | -1.00 | 0.32 | -16.51 | 5.34 |
| m_pcr_infected | ~ | Ga | -4.24 | 6.37 | -0.67 | 0.51 | -16.73 | 8.24 |
| m_pcr_infected | ~ | En | -0.22 | 3.25 | -0.07 | 0.95 | -6.60 | 6.15 |
| m_pcr_parasite_count | ~ | So1 | -10.98 | 4.35 | -2.53 | 0.01 | -19.50 | -2.46 |
| m_pcr_parasite_count | ~ | Ga | -1.90 | 4.97 | -0.38 | 0.70 | -11.63 | 7.84 |
| m_pcr_parasite_count | ~ | En | -3.52 | 2.54 | -1.39 | 0.17 | -8.50 | 1.45 |
| m_HL_ratio_0ifna | ~ | m_pcr_infected | -0.07 | 0.04 | -1.49 | 0.14 | -0.15 | 0.02 |
| m_HL_ratio_0ifna | ~ | m_pcr_parasite_count | 0.04 | 0.05 | 0.81 | 0.42 | -0.06 | 0.15 |
| m_HL_ratio_0ifna | ~ | p3_asymmetry | 0.24 | 6.81 | 0.04 | 0.97 | -13.11 | 13.59 |
| m_leucocytes_0ifna | ~ | m_pcr_infected | -0.08 | 0.16 | -0.51 | 0.61 | -0.41 | 0.24 |
| m_leucocytes_0ifna | ~ | m_pcr_parasite_count | 0.16 | 0.20 | 0.82 | 0.42 | -0.23 | 0.56 |
| m_leucocytes_0ifna | ~ | p3_asymmetry | 24.15 | 25.33 | 0.95 | 0.34 | -25.50 | 73.80 |
| m_pcr_infected | ~~ | m_pcr_parasite_count | 0.12 | 0.03 | 4.73 | 0.00 | 0.07 | 0.17 |
| m_HL_ratio_0ifna | ~~ | m_leucocytes_0ifna | 0.00 | 0.01 | -0.65 | 0.52 | -0.02 | 0.01 |
| m_pcr_infected | ~~ | m_pcr_infected | 0.21 | 0.04 | 5.83 | 0.00 | 0.14 | 0.29 |
| m_pcr_parasite_count | ~~ | m_pcr_parasite_count | 0.13 | 0.02 | 5.83 | 0.00 | 0.09 | 0.17 |
| m_HL_ratio_0ifna | ~~ | m_HL_ratio_0ifna | 0.02 | 0.00 | 5.83 | 0.00 | 0.01 | 0.02 |
| m_leucocytes_0ifna | ~~ | m_leucocytes_0ifna | 0.21 | 0.04 | 5.83 | 0.00 | 0.14 | 0.28 |
| So1 | ~~ | So1 | 0.00 | 0.00 | NA | NA | 0.00 | 0.00 |
| So1 | ~~ | Ga | 0.00 | 0.00 | NA | NA | 0.00 | 0.00 |
| So1 | ~~ | En | 0.00 | 0.00 | NA | NA | 0.00 | 0.00 |
| So1 | ~~ | p3_asymmetry | 0.00 | 0.00 | NA | NA | 0.00 | 0.00 |
| Ga | ~~ | Ga | 0.00 | 0.00 | NA | NA | 0.00 | 0.00 |
| Ga | ~~ | En | 0.00 | 0.00 | NA | NA | 0.00 | 0.00 |
| Ga | ~~ | p3_asymmetry | 0.00 | 0.00 | NA | NA | 0.00 | 0.00 |
| En | ~~ | En | 0.00 | 0.00 | NA | NA | 0.00 | 0.00 |
| En | ~~ | p3_asymmetry | 0.00 | 0.00 | NA | NA | 0.00 | 0.00 |
| p3_asymmetry | ~~ | p3_asymmetry | 0.00 | 0.00 | NA | NA | 0.00 | 0.00 |
|  |  |  |  |  |  |  |  |  |
| ***F. coelebs, primary 3 asymmetry (hypothesis 1a, model name 1a7); Fig. S4c*** | | | | | | | | |
| **Pathway** | | | **Estimate** | **SE** | **z** | **P-value** | **CI, lower** | **CI, upper** |
| m_pcr_infected | ~ | So1 | -0.38 | 4.10 | -0.09 | 0.93 | -8.41 | 7.65 |
| m_pcr_infected | ~ | Ga | 2.58 | 3.75 | 0.69 | 0.49 | -4.77 | 9.92 |
| m_pcr_infected | ~ | En | 3.69 | 2.77 | 1.33 | 0.18 | -1.74 | 9.11 |
| m_pcr_parasite_count | ~ | So1 | -12.94 | 9.71 | -1.33 | 0.18 | -31.97 | 6.09 |
| m_pcr_parasite_count | ~ | Ga | 11.24 | 8.88 | 1.27 | 0.21 | -6.17 | 28.65 |
| m_pcr_parasite_count | ~ | En | 6.44 | 6.56 | 0.98 | 0.33 | -6.41 | 19.29 |
| m_HL_ratio_0ifna | ~ | m_pcr_infected | 0.01 | 0.13 | 0.10 | 0.92 | -0.24 | 0.26 |
| m_HL_ratio_0ifna | ~ | m_pcr_parasite_count | 0.09 | 0.05 | 1.72 | 0.09 | -0.01 | 0.19 |
| m_HL_ratio_0ifna | ~ | p3_asymmetry | 17.09 | 13.60 | 1.26 | 0.21 | -9.56 | 43.74 |
| m_leucocytes_0ifna | ~ | m_pcr_infected | 0.26 | 0.24 | 1.08 | 0.28 | -0.21 | 0.72 |
| m_leucocytes_0ifna | ~ | m_pcr_parasite_count | -0.15 | 0.10 | -1.49 | 0.14 | -0.34 | 0.05 |
| m_leucocytes_0ifna | ~ | p3_asymmetry | -23.17 | 25.61 | -0.90 | 0.37 | -73.37 | 27.03 |
| m_pcr_infected | ~~ | m_pcr_parasite_count | 0.10 | 0.04 | 2.91 | 0.00 | 0.03 | 0.17 |
| m_HL_ratio_0ifna | ~~ | m_leucocytes_0ifna | -0.03 | 0.01 | -2.03 | 0.04 | -0.05 | 0.00 |
| m_pcr_infected | ~~ | m_pcr_infected | 0.08 | 0.02 | 4.53 | 0.00 | 0.05 | 0.12 |
| m_pcr_parasite_count | ~~ | m_pcr_parasite_count | 0.47 | 0.10 | 4.53 | 0.00 | 0.27 | 0.67 |
| m_HL_ratio_0ifna | ~~ | m_HL_ratio_0ifna | 0.04 | 0.01 | 4.53 | 0.00 | 0.02 | 0.06 |
| m_leucocytes_0ifna | ~~ | m_leucocytes_0ifna | 0.15 | 0.03 | 4.53 | 0.00 | 0.08 | 0.21 |
| So1 | ~~ | So1 | 0.00 | 0.00 | NA | NA | 0.00 | 0.00 |
| So1 | ~~ | Ga | 0.00 | 0.00 | NA | NA | 0.00 | 0.00 |
| So1 | ~~ | En | 0.00 | 0.00 | NA | NA | 0.00 | 0.00 |
| So1 | ~~ | p3_asymmetry | 0.00 | 0.00 | NA | NA | 0.00 | 0.00 |
| Ga | ~~ | Ga | 0.00 | 0.00 | NA | NA | 0.00 | 0.00 |
| Ga | ~~ | En | 0.00 | 0.00 | NA | NA | 0.00 | 0.00 |
| Ga | ~~ | p3_asymmetry | 0.00 | 0.00 | NA | NA | 0.00 | 0.00 |
| En | ~~ | En | 0.00 | 0.00 | NA | NA | 0.00 | 0.00 |
| En | ~~ | p3_asymmetry | 0.00 | 0.00 | NA | NA | 0.00 | 0.00 |
| p3_asymmetry | ~~ | p3_asymmetry | 0.00 | 0.00 | NA | NA | 0.00 | 0.00 |
|  |  |  |  |  |  |  |  |  |
| ***P. major, primary 3 asymmetry (hypothesis 2a, model name 2a5); Fig. S4d*** | | | | | | | | |
| **Pathway** | | | **Estimate** | **SE** | **z** | **P-value** | **CI, lower** | **CI, upper** |
| p3_asymmetry | ~ | So1 | -0.05 | 0.03 | -1.95 | 0.05 | -0.10 | 0.00 |
| p3_asymmetry | ~ | Ga | 0.04 | 0.03 | 1.34 | 0.18 | -0.02 | 0.09 |
| p3_asymmetry | ~ | En | 0.00 | 0.01 | 0.04 | 0.97 | -0.03 | 0.03 |
| m_pcr_parasite_count | ~ | p3_asymmetry | 7.64 | 29.96 | 0.26 | 0.80 | -51.08 | 66.36 |
| m_HL_ratio_0ifna | ~ | m_pcr_parasite_count | -0.05 | 0.03 | -1.54 | 0.12 | -0.11 | 0.01 |
| m_leucocytes_0ifna | ~ | m_pcr_parasite_count | -0.06 | 0.08 | -0.76 | 0.45 | -0.23 | 0.10 |
| m_pcr_parasite_count | ~~ | m_pcr_infected | 0.15 | 0.03 | 4.72 | 0.00 | 0.09 | 0.22 |
| m_HL_ratio_0ifna | ~~ | m_leucocytes_0ifna | 0.02 | 0.01 | 1.81 | 0.07 | 0.00 | 0.04 |
| p3_asymmetry | ~~ | p3_asymmetry | 0.00 | 0.00 | 6.36 | 0.00 | 0.00 | 0.00 |
| m_pcr_parasite_count | ~~ | m_pcr_parasite_count | 0.45 | 0.07 | 6.36 | 0.00 | 0.31 | 0.59 |
| m_HL_ratio_0ifna | ~~ | m_HL_ratio_0ifna | 0.03 | 0.01 | 6.36 | 0.00 | 0.02 | 0.04 |
| m_leucocytes_0ifna | ~~ | m_leucocytes_0ifna | 0.26 | 0.04 | 6.36 | 0.00 | 0.18 | 0.34 |
| m_pcr_infected | ~~ | m_pcr_infected | 0.14 | 0.02 | 6.36 | 0.00 | 0.09 | 0.18 |
| So1 | ~~ | So1 | 0.00 | 0.00 | NA | NA | 0.00 | 0.00 |
| So1 | ~~ | Ga | 0.00 | 0.00 | NA | NA | 0.00 | 0.00 |
| So1 | ~~ | En | 0.00 | 0.00 | NA | NA | 0.00 | 0.00 |
| Ga | ~~ | Ga | 0.00 | 0.00 | NA | NA | 0.00 | 0.00 |
| Ga | ~~ | En | 0.00 | 0.00 | NA | NA | 0.00 | 0.00 |
| En | ~~ | En | 0.00 | 0.00 | NA | NA | 0.00 | 0.00 |
|  |  |  |  |  |  |  |  |  |
| ***S. atricapilla, primary 3 asymmetry (hypothesis 1a, model name 1a5); Fig. S4e*** | | | | | | | | |
| **Pathway** | | | **Estimate** | **SE** | **z** | **P-value** | **CI, lower** | **CI, upper** |
| m_pcr_parasite_count | ~ | So1 | -11.34 | 9.57 | -1.18 | 0.24 | -30.09 | 7.42 |
| m_pcr_parasite_count | ~ | Ga | 20.33 | 5.78 | 3.52 | 0.00 | 9.00 | 31.65 |
| m_pcr_parasite_count | ~ | En | -4.65 | 3.83 | -1.22 | 0.22 | -12.16 | 2.85 |
| m_HL_ratio_0ifna | ~ | m_pcr_parasite_count | -0.01 | 0.04 | -0.25 | 0.80 | -0.10 | 0.07 |
| m_leucocytes_0ifna | ~ | m_pcr_parasite_count | 0.16 | 0.09 | 1.83 | 0.07 | -0.01 | 0.34 |
| p3_asymmetry | ~ | m_HL_ratio_0ifna | 0.00 | 0.00 | -1.71 | 0.09 | -0.01 | 0.00 |
| p3_asymmetry | ~ | m_leucocytes_0ifna | 0.00 | 0.00 | 1.04 | 0.30 | 0.00 | 0.00 |
| m_pcr_parasite_count | ~~ | m_pcr_infected | 0.08 | 0.02 | 3.59 | 0.00 | 0.04 | 0.12 |
| m_HL_ratio_0ifna | ~~ | m_leucocytes_0ifna | -0.01 | 0.01 | -0.54 | 0.59 | -0.02 | 0.01 |
| m_pcr_parasite_count | ~~ | m_pcr_parasite_count | 0.29 | 0.06 | 5.20 | 0.00 | 0.18 | 0.40 |
| m_HL_ratio_0ifna | ~~ | m_HL_ratio_0ifna | 0.04 | 0.01 | 5.20 | 0.00 | 0.02 | 0.05 |
| m_leucocytes_0ifna | ~~ | m_leucocytes_0ifna | 0.15 | 0.03 | 5.20 | 0.00 | 0.09 | 0.20 |
| p3_asymmetry | ~~ | p3_asymmetry | 0.00 | 0.00 | 5.20 | 0.00 | 0.00 | 0.00 |
| m_pcr_infected | ~~ | m_pcr_infected | 0.07 | 0.01 | 5.20 | 0.00 | 0.04 | 0.09 |
| So1 | ~~ | So1 | 0.00 | 0.00 | NA | NA | 0.00 | 0.00 |
| So1 | ~~ | Ga | 0.00 | 0.00 | NA | NA | 0.00 | 0.00 |
| So1 | ~~ | En | 0.00 | 0.00 | NA | NA | 0.00 | 0.00 |
| Ga | ~~ | Ga | 0.00 | 0.00 | NA | NA | 0.00 | 0.00 |
| Ga | ~~ | En | 0.00 | 0.00 | NA | NA | 0.00 | 0.00 |
| En | ~~ | En | 0.00 | 0.00 | NA | NA | 0.00 | 0.00 |
|  |  |  |  |  |  |  |  |  |
| ***T. merula, primary 3 asymmetry (hypothesis 2a, model name 2a4); Fig. S4f*** | | | | | | | | |
| **Pathway** | | | **Estimate** | **SE** | **z** | **P-value** | **CI, lower** | **CI, upper** |
| p3_asymmetry | ~ | So1 | 0.00 | 0.02 | 0.00 | 1.00 | -0.05 | 0.05 |
| p3_asymmetry | ~ | Ga | -0.01 | 0.03 | -0.50 | 0.62 | -0.06 | 0.04 |
| p3_asymmetry | ~ | En | -0.02 | 0.02 | -1.14 | 0.25 | -0.05 | 0.01 |
| m_pcr_infected | ~ | p3_asymmetry | 9.78 | 9.78 | 1.00 | 0.32 | -9.39 | 28.94 |
| m_HL_ratio_0ifna | ~ | m_pcr_infected | 0.15 | 0.20 | 0.74 | 0.46 | -0.24 | 0.53 |
| m_leucocytes_0ifna | ~ | m_pcr_infected | -0.60 | 0.33 | -1.79 | 0.07 | -1.25 | 0.06 |
| m_pcr_infected | ~~ | m_pcr_parasite_count | 0.01 | 0.01 | 1.29 | 0.20 | -0.01 | 0.03 |
| m_HL_ratio_0ifna | ~~ | m_leucocytes_0ifna | -0.01 | 0.01 | -0.69 | 0.49 | -0.02 | 0.01 |
| p3_asymmetry | ~~ | p3_asymmetry | 0.00 | 0.00 | 5.00 | 0.00 | 0.00 | 0.00 |
| m_pcr_infected | ~~ | m_pcr_infected | 0.02 | 0.00 | 5.00 | 0.00 | 0.01 | 0.03 |
| m_HL_ratio_0ifna | ~~ | m_HL_ratio_0ifna | 0.04 | 0.01 | 5.00 | 0.00 | 0.02 | 0.05 |
| m_leucocytes_0ifna | ~~ | m_leucocytes_0ifna | 0.11 | 0.02 | 5.00 | 0.00 | 0.07 | 0.15 |
| m_pcr_parasite_count | ~~ | m_pcr_parasite_count | 0.23 | 0.05 | 5.00 | 0.00 | 0.14 | 0.32 |
| So1 | ~~ | So1 | 0.00 | 0.00 | NA | NA | 0.00 | 0.00 |
| So1 | ~~ | Ga | 0.00 | 0.00 | NA | NA | 0.00 | 0.00 |
| So1 | ~~ | En | 0.00 | 0.00 | NA | NA | 0.00 | 0.00 |
| Ga | ~~ | Ga | 0.00 | 0.00 | NA | NA | 0.00 | 0.00 |
| Ga | ~~ | En | 0.00 | 0.00 | NA | NA | 0.00 | 0.00 |
| En | ~~ | En | 0.00 | 0.00 | NA | NA | 0.00 | 0.00 |

# ^1^ Model name: The first number refers to whether the model is amended with a measure for vector abundance (1: original model, without measure for vector abundance, 2: model including measure for vector abundance). The first letter describes the underlying hypothesis (a: 'top-down'-hypothesis, b: 'bottom-up'-hypothesis, see Background). The number following the letter indicates the model number (not further described). The addition of an 'x' indicated whether that model was modelled using the species-specific parasite-vector relationship (i.e. *Plasmodium/Haemoproteus* infection in relation the abundance of vectors of the families Culicidae/Ceratopogonidae). E.g.: 1b11x indicates the original model (1), model number (11), 'bottom-up'-hypothesis (addition of 'x').

**Table S6.** Overview of sampled plots with regards to protection status.

| **Forest category** | **Plot ID** | **Conservation Type** | **German** | **English** |
| --- | --- | --- | --- | --- |
| Unmanaged | SEW45 | LSG | *Landschaftschutzgebiet* | Landscape conservation area |
| Unmanaged | SEW46 | FFH | *Fauna-Flora-Habitat-Richlinie* | Habitats Directive |
| Unmanaged | SEW47 | LSG | *Landschaftschutzgebiet* | Landscape conservation area |
| Unmanaged | SEW48 | LSG | *Landschaftschutzgebiet* | Landscape conservation area |
| Unmanaged | SEW7 | LSG | *Landschaftschutzgebiet* | Landscape conservation area |
| Unmanaged | SEW8 | LSG | *Landschaftschutzgebiet* | Landscape conservation area |
| Unmanaged | SEW9 | LSG | *Landschaftschutzgebiet* | Landscape conservation area |
| Age Class: Old | SEW35 | LSG | *Landschaftschutzgebiet* | Landscape conservation area |
| Age Class: Old | SEW40 | LSG | *Landschaftschutzgebiet* | Landscape conservation area |
| Age Class: Old | SEW43 | LSG | *Landschaftschutzgebiet* | Landscape conservation area |
| Age Class: Old | SEW44 | NSG1 | *Bundesnaturschutzgesetz* | German Federal Nature Conservation Act (1) |
| Age Class: Old | SEW5 | NSG1 | *Bundesnaturschutzgesetz* | German Federal Nature Conservation Act (1) |
| Age Class: Old | SEW50 | NSG1 | *Bundesnaturschutzgesetz* | German Federal Nature Conservation Act (1) |
| Age Class: Old | SEW6 | NSG1 | *Bundesnaturschutzgesetz* | German Federal Nature Conservation Act (1) |
| Age Class: Young | SEW36 | LSG | *Landschaftschutzgebiet* | Landscape conservation area |
| Age Class: Young | SEW37 | LSG | *Landschaftschutzgebiet* | Landscape conservation area |
| Age Class: Young | SEW38 | NSG2 | *Bundesnaturschutzgesetz* | German Federal Nature Conservation Act (2) |
| Age Class: Young | SEW39 | LSG | *Landschaftschutzgebiet* | Landscape conservation area |
| Age Class: Young | SEW41 | NSG1 | *Bundesnaturschutzgesetz* | German Federal Nature Conservation Act (1) |
| Age Class: Young | SEW42 | NSG1 | *Bundesnaturschutzgesetz* | German Federal Nature Conservation Act (1) |
| Age Class: Young | SEW49 | NSG1 | *Bundesnaturschutzgesetz* | German Federal Nature Conservation Act (1) |

# Additional file 1: References cited in Addition file 1

1. van Hoesel W, Marzal A, Magallanes S, Santiago-Alarcon D, Ibáñez-Bernal S, Renner SC: **Management of ecosystems alters vector dynamics and haemosporidian infections**. *Scientific Reports* 2019, **9**(1):8779.
